# Ecological Factors Influencing the Occurrence of Macrofungi from Eastern Mountainous Areas to the Central Plains of Jilin Province, China

**DOI:** 10.3390/jof8080871

**Published:** 2022-08-18

**Authors:** Jia-Jun Hu, Gui-Ping Zhao, Yong-Lan Tuo, Zheng-Xiang Qi, Lei Yue, Bo Zhang, Yu Li

**Affiliations:** 1 Engineering Research Centre of Edible and Medicinal Fungi, Ministry of Education, Jilin Agricultural University, Changchun 130118, China; 2 School of Life Science, Northeast Normal University, Changchun 130024, China; 3 Joint Laboratory of International Cooperation in Modern Agricultural Technology, Ministry of Education, Jilin Agricultural University, Changchun 130118, China; 4 China Mycological Valley (Hefei), Hefei 231100, China

**Keywords:** ecology, environmental factors, forest type, forest health, landform, macrofungi occurrence

## Abstract

Macrofungi are essential in forest ecological functioning. Their distribution and diversity are primarily impacted by vegetation, topography, and environmental factors, such as precipitation and temperature. However, the composition and topographical changes of the macrofungi between the eastern mountainous area and central plains of Jilin Province are currently unknown. For this study, we selected six investigational sites representing three different topographical research sites in Jilin Province to assess macrofungal diversity, and applied a quadrat sampling method. Macro- and micro-morphological characteristics combined with the molecular method were used to identify the collected macrofungi. Meanwhile, selected meteorological data were obtained for statistical analysis. As a result, 691 species were identified, of which Agarics were the most common, accounting for 60.23%, while the Cantharelloid fungi were the least common (0.91%). Furthermore, most of the shared genera (species) were saprophytic. The α diversity showed that the species diversity and richness in Longwan National Forest Park (B2) were the highest at the genus level. The mycorrhizal macrofungi proportion revealed that Quanshuidong Forest Farm (A1) was the healthiest. Finally, species composition similarity decreased with the transition from mountainous to hilly plains. We concluded that the occurrence of macrofungi was most influenced by vegetation. The air humidity, precipitation, and wind velocity were also found to significantly impact the occurrence of macrofungi. Finally, the mycorrhizal:saprophytic ratios and species similarity decreased with the transition from the mountainous area to the plains. The results presented here help elucidate the macrofungi composition and their relationship with environmental factors and topography in Jilin Province, which is crucial for sustainable utilization and future conservation.

## 1. Introduction

Human beings have discovered and utilized macrofungi throughout history with an awareness of the essential role in forest ecology. Macrofungi form mycorrhizal symbioses with host plants to promote the absorbance of substances, such as mineral elements and water. They also improve the tolerance of host plants to heavy metals, promote the survival and growth of afforestation and seedlings, and improve the diversity and stability of plants in the forest ecosystem. The comprehensive effects of macrofungi on the forest ecosystem are mainly manifested by increasing the plant–soil connections, improving the soil structure, promoting soil microorganisms, enhancing the function of plant organs, resisting antagonistic plant root disease pathogens, and degrading wood and other substances [1,2,3,4]. Saprotrophic macrofungi are also involved in the material cycle and energy flow, such as decomposing fallen timber and dead wood into other substances, such as lignin, cellulose, and hemicellulose [5,6], finally converted into glucose, fructose, etc.

Forests, as a habitat, are essential for the growth of macrofungi. Studies have shown that canopy openness, vegetation structure, and tree species richness strongly influence the occurrence of macrofungal functional groups [7]. Plant size, tree density, herb richness, and evenness could also affect the macrofungi composition [8]. Wood rotting fungi are strongly associated with tree species and the degree of wood decay [9]. Additionally, the species richness of macrofungi is also significantly related to forest management [10,11,12].

Topography is an abiotic factor that affects macrofungi community structures, as spatial eigenvectors—for example, slope—strongly connect with macrofungi occurrence [13]. Topography, more importantly, forms micro-habitats, thus creating differences in the key factors such as temperature, air humidity, and light, which affect the occurrence of macrofungi.

Furthermore, the occurrence and growth of macrofungi are closely related to environmental factors. For example, ectomycorrhizal macrofungi (EM fungi) are closely associated with their host plants, as Tuo et al. revealed the quantity of EM fungi in Wunvfeng National Forest Park, China, was positively correlated with the occurrence of *Quercus mongolica* [6]. Humidity is another critical factor, and Trudell et al. characterized the epigeous macrofungi communities in two old-growth conifer forests with a high level of similarity in their dominant tree species and proposed that the differences between the macrofungi communities were primarily related to the disparities in ecosystem moisture [14]. In addition, pH [15], soil temperature [16], and organic matter content [17,18] could also affect the occurrence of macrofungi.

There are apparent differences in the topography of Jilin Province. The terrain inclines from southeast to northwest and could be divided into two significant landforms: the eastern mountainous area and the central-western plains. The eastern mountainous area comprises the middle part of Mt. Changbai and its branches [19], accounting for 33% of the total area; hills account for 6% of the area. Due to the unique natural geographical environment and meteorological conditions, Jilin Province has become one of China’s biodiversity hotspots. New macrofungi species have been discovered here, including *Cortinarius laccariphyllus* Y. Li and M.L. Xie; *Cortinarius neotorvus* Y. Li, M.L. Xie, and T.Z. Wei [20]; *Cordyceps changchunensis* J.J. Hu, Bo Zhang, and Y. Li; *Cordyceps changbaiensis* J.J. Hu, Bo Zhang, and Y. Li; and *Cordyceps jingyuetanensis* J.J. Hu, Bo Zhang, and Y. Li [21]. However, the macrofungi distribution patterns and the relationship with the environmental factors are currently unclear.

With this study, we aim to understand the species composition and macrofungal distribution in the central and eastern areas of Jilin Province and analyze the relationship between their occurrence and environmental factors. In addition, we analyze the changes in the macrofungi composition about to topography.

## 2. Materials and Methods

### 2.1. Introduction of Investigation Site

Jilin Province has a temperate continental monsoon climate, with four distinct seasons, rain and heat in the same season, noticeable seasonal changes, and regional differences in temperature and precipitation. The average temperature is below −11 °C in the winter and above 23 °C in the summer [22,23,24], and the average annual precipitation is 400–600 mm [25,26,27]. However, precipitation significantly differs between seasons and regions, as 80% is concentrated in the summer, and the eastern area is the richest.

**Figure 1:**
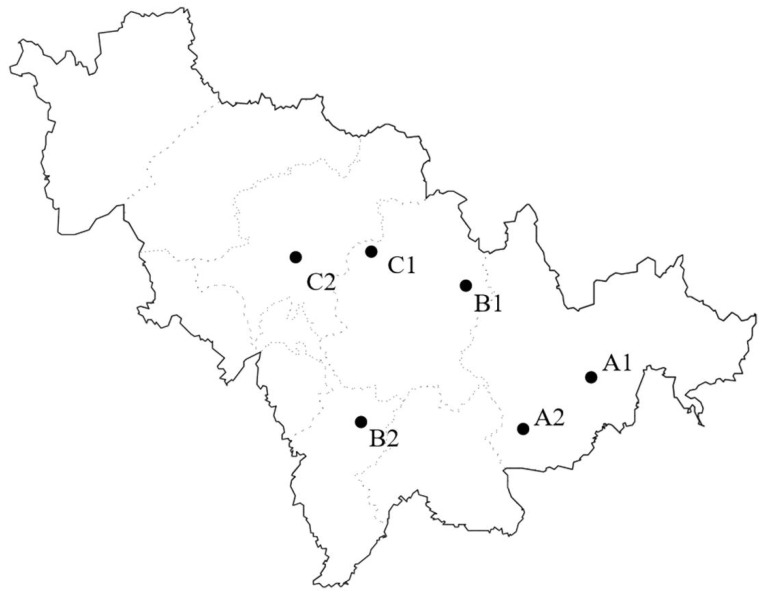
Distribution map showing the investigation sites in Jilin Province (A1: Quanshuidong Forest Farm; A2: Lushuihe National Forest Park; B1: Shengli River Forest Farm; B2: Longwan National Forest Park; C1: Zuojia Region; C2: Jingyuetan National Forest Park).

**Figure 2:**
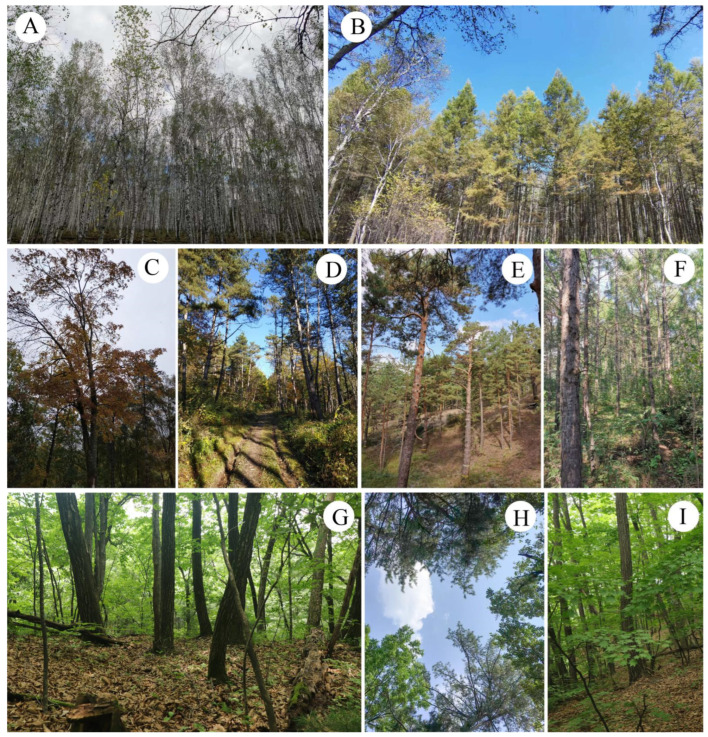
Forest types from the investigation sites in Jilin Province. (**A**) *Betula platyphylla* forest; (**B**) *Cunninghamia* forest; (**C**) broadleaf mixed forest (mainly *Acer* sp.); (**D**–**F**) *Pine* forest; (**G**) *Quercus mongolica* forest; (**H**) Coniferous and broadleaf mixed forest (*Pinus* and broadleaf mixed forest); (**I**) *Quercus mongolica* forest.

Six representative investigation sites were selected to assess the macrofungi resources and analyze the relationships between macrofungi occurrence in eastern Jilin Province, China, in detail (Figure 1 and Figure 2 and Table 1). These sites are Mt. Changbai area (A)—Quanshuidong Forest Farm (A1) and Lushuihe National Forest Park (A2); Mt. Changbai branches, Mt. Laoyeling branch (B)—Shengli River Forest Farm (B1) and Mt. Longgang branch—Longwan National Forest Park (B2); and low hilly plain areas (C)—Zuojia Region (C1) and Jingyuetan National Forest Park (C2). The investigation also aimed to improve our understanding of the composition transitions from the eastern to central regions, which cause changes in topography, vegetation, precipitation, and temperature.

(1) Quanshuidong Forest Farm (A1)

Quanshuidong Forest Farm, located in Helong City, Jilin Province, belongs to the mid-temperate monsoonal semi-humid climate zone. The annual average temperature is 5.6 °C, and the effective accumulated temperature at 10 °C is 2534.0 °C. The average yearly precipitation is 573.6 mm, and the frost-free period is approximately 138 days [28].

(2) Lushuihe National Forest Park (A2)

Lushuihe National Forest Park, located in Fusong County, Baishang City, Jilin Province, belongs to the temperate monsoon climate. The annual average temperature is 2.9 °C, and the effective accumulated temperature at 10 °C is 2606.9 °C. The average yearly precipitation is 894 mm, and the frost-free period is approximately 110 days [29].

(3) Laoyeling Branch—Shengli River Forest Farm (B1)

Mt. Laoyeling belongs to the Mt. Changbai Systems and is in the northeast–southwest direction, 800–1000 m above sea level in Jilin Province, with a relative height of approximately 500 m. The landforms are mainly low and middle mountains, with narrow valleys between the mountains. Volcanoes and lava flows are widely distributed in this area [27].

Shengli River Forest Farm has a temperate continental climate with an average annual temperature of 3.8 °C, an average annual rainfall of 633.7 mm, and a frost-free period of 110–130 days.

(4) Longgang Mountain Branch—Longwan National Forest Park (B2)

Longwan National Forest Park is in the middle section of Mt. Longgang in Huinan County, Jilin Province, with an average sea level of 880 m. It has a northern temperate continental monsoon climate. The annual average temperature is 4.8 °C, the minimum temperature is −17 °C, and the maximum monthly average temperature is 22.4 °C. The sufficient accumulated temperature at 10 °C is 2728 °C. The average annual precipitation is 837.9 mm, ranging from 436.5 to 987.2 mm. The maximum daily precipitation is 124.2 mm, concentrated from June to August, with an average yearly frost-free period of 138 days and an average sunshine time of 2296 h [30].

(5) Zuojia Region (C1)

The Zuojia area, Jilin Province, belongs to the hilly plain areas of Mt. Changbai. It has a continental climate with temperate monsoons and often experiences Siberian cold waves, with changeable weather and distinct seasons. The annual average temperature is 5.6 °C, and the effective accumulated temperature at 10 °C is 2779.8 °C. The average yearly precipitation is 679 mm, the average annual evaporation is 1200 mm, and the frost-free period is approximately 120 days [31,32,33].

(6) Jingyuetan National Forest Park (C2)

Jingyuetan National Forest Park is in the transitional zone from the eastern mountain area to the western grasslands of Jilin Province. It belongs to the hill areas of Mt. Changbai. The elevation is between 245.8 and 371.6 m above sea level. The climate is temperate semi-dry, early, and semi-humid monsoon with four distinct seasons. The annual average temperature is 6.1 °C. The average annual precipitation is 577.3 mm. The rainy season is mainly concentrated in July and August, as it accounts for 67% of the annual precipitation, the annual evaporation is 1392.5 mm, and there is a frost-free period of 145 d [34].

### 2.2. Macrofungi Investigation

(1) Investigation

In this study, four plots were selected at each investigation site in which three quadrats of 10 m × 10 m [35] were set in parallel at each plot, and each plot was numbered with a distance of 300 m, applied with a quadrat sampling method.

(2) Specimen collection and recording

**Figure 3:**
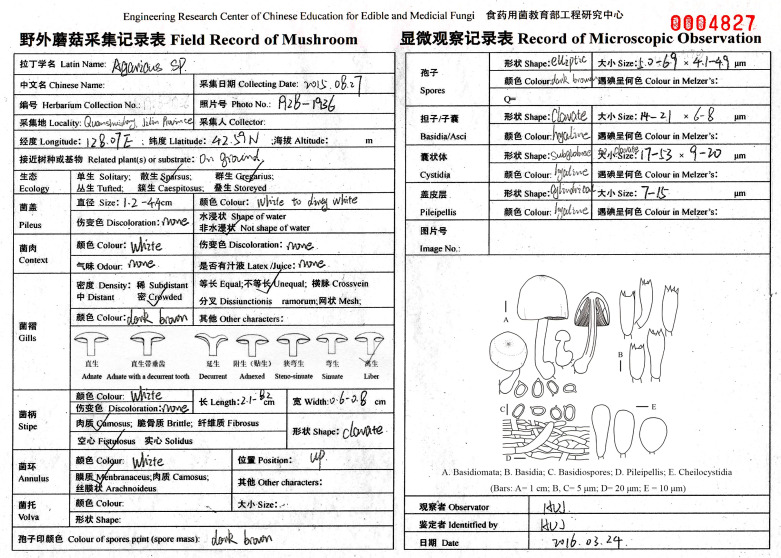
An example of field record and microscopic observation record of the collected specimen.

Specimens were mainly collected from July to September at every investigation site. The specimens were photographed in situ. The habitat, altitude, soil characteristics, and nearby trees were recorded. The size of basidiomata was measured when fresh, and features such as striations, hygrophanous, and squama were noted (Figure 3). After examining and describing the fresh macroscopic characters, the specimens were dried in an electric drier at 45–50 °C. All the collected specimens had conspicuous basidiomata.

(3) Specimen identification

The dried specimens were rehydrated in 94% ethanol for microscopic examination, then mounted in 3% potassium hydroxide (KOH), 1% Congo red, and Melzer’s reagent; they were then examined with a Zeiss Axiolab A1 microscope (Carl Zeiss, Jena, Germany) at magnifications up to 1000×. All measurements were taken from the sections mounted in 1% Congo red. A minimum of 40 spores, 20 basidia/asci, 20 cystidia, etc., were measured from at least two different fruiting bodies for each specimen [36]. When combined with the macroscopic characteristics, the classification status of the specimens was determined by referring to literature and monographs [37]. For some species, we also sequenced, and the sequences have been deposited in GenBank (GenBank accession numbers: ON683416–ON683495). The taxonomic status of all species is referenced in the Index Fungorum [38]. The specimens examined were deposited in the Herbarium of Mycology of Jilin Agricultural University (HMJAU).

(4) Data collection

The meteorological data—average temperature (T), average relative humidity (RH), average monthly precipitation (P), average wind speed, and accumulated temperature from July to September (AT)—were downloaded from the China meteorological data network [39]. The soil type and representative forest type data were obtained from the Chinese Academy of Sciences (Table 2) [40].

### 2.3. Data Analysis

Two alpha diversity indices, the Simpson diversity index [41] and the Shannon–Wiener index [42], were calculated at the genus level for each investigation site to analyze the community composition of the macrofungi. The Shannon index (H′) reflected the diversity of the community species. The Simpson index (D) reflected the probability of two individuals being randomly selected from the same sample, and these two individuals are from the same class.

The compositions of the macrofungi at Mt. Changbai (A), the transitional zone (B), and the plain hilly area (C) were compared by calculating the similarity coefficient (S) [43] and generating a complex heatmap [44]. The macrofungi compositions between Mt. Changbai and Mt. Laoyeling branch and Mt. Longgang Branch were also compared.

The diversity index formulae were as follows:

(1) $$H^{\prime }=-\sum Pi\ln\left(Pi\right)$$

(2) $$D=1-\sum Pi^{2}$$

(3) $$S=\frac{2a}{b+c}\times 100$$

where *Pi* is the proportion of species *i* to the total number of individuals of all species in the plot, *a* is the number of genera shared by the two places, and *b* and *c* are the genera that appear in the same place.

According to the *Atlas of Chinese Macrofungal Resources* [37], identified species were divided into eight categories: Larger Ascomycete, Agarics, Polyporoid, Hyonaceous and Thelephoroid fungi (PHT fungi), Cantharelloid fungi, Gasteroid fungi, Jelly fungi, Coral fungi, and Boletes.

The identified genera were summarized in an Excel table. Then, we specified a value of 0/1 for each genus shown at each investigation site (0 means that the genus did not appear at the investigation site). Originpro 2019 (OriginLab, Northampton, USA) was used to analyze common genera. Dominant family (number of species more than ten of the family) and dominant genus (number of species more than five of the genera) of each investigation site were counted [45]. Moreover, the species numbers per family (genera) at all six investigation sites were statistics, the top ten families (genera) were shown, and the bubble matrix was drowned.

The software Canoco 5.0 (Micro-computer Power, Ithaca, NY, USA) [46] fits and analyzes the relationship between macrofungi species and the environmental factors at six investigation sites. Log (*n* + 1) was used to reduce meteorological data quality and balance the vast differences among the various factors. The quadrat × species matrix and the quadrat × environment matrix were established. Two-dimensional sorting and canonical correspondence analysis (CCA) of macrofungi and environmental factors in six different vegetation types were conducted.

## 3. Results

### 3.1. Composition Characteristics of the Macrofungi

**Figure 4:**
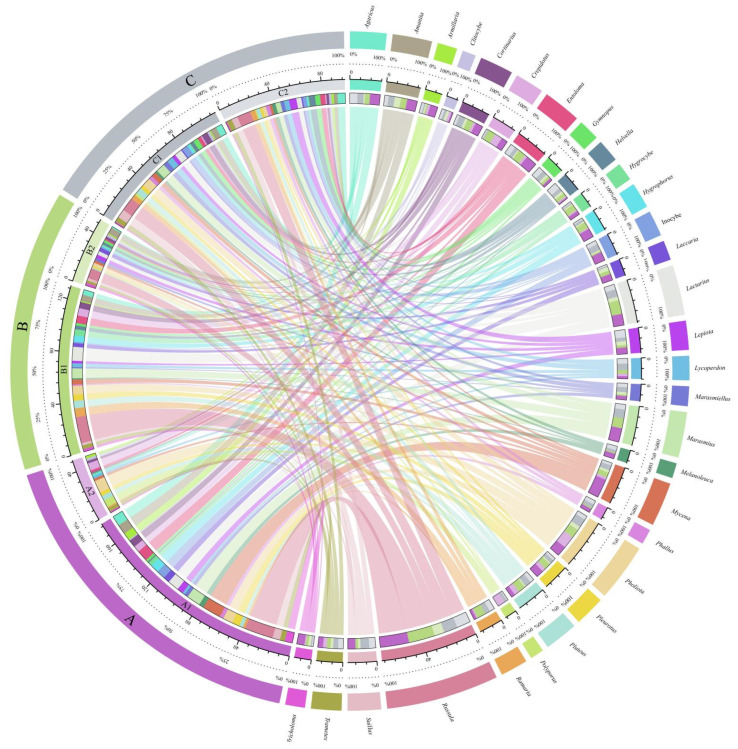
Circos plot shows the relative abundance of macrofungi in six different sites in Jinlin Province based on the genera with more than four species. The analysis of species abundance shows *Russula* was the common genus and site A1 contains more species than the other five investigation sites. A1: Quanshuidong Forest Farm; A2: Lushuihe National Forest Form; B1: Shengli River Forest Farm; B2: Longwan National Forest Park; C1: Zuojia Region; C2: Jingyuetan National Forest Park.

In this study, genera with more than four species were chosen from the six investigation sites for analysis. Site A1 included the most identified families and species among six investigation sites, while site B2 had the fewest (Figure 4). General overview, *Russula* Pers., *Pholiota* (Fr.) P. Kumm., and *Mycena* (Pers.) Roussel, etc., were the most common genera recorded. Taxa belonging to *Russula* and *Mycena* were the most common at site A1; *Pholiota* and *Polyporus* were the most common ones at site A2; *Russula*, *Pholiota*, and *Hygrophorus* Fr. were the most reported genera at site B1; *Russula* and *Amanita* Pers were the most recorded at site B2; *Russula*, *Suillus* Gray, and *Pholiota* were the most reported at site C1; *Russula*, *Suillus*, and *Agaricus* L., were the most common genera at site C2. Overall, the most reported one is *Russula*.

### 3.2. Shared Genera (Species) Analysis

**Figure 5:**
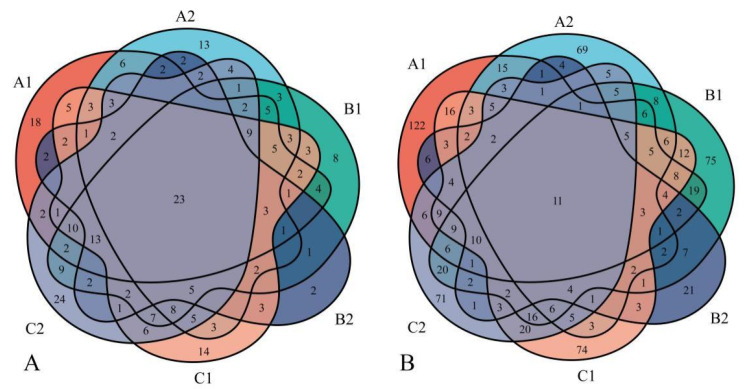
The co-occurring genera and species were analyzed in six different investigation sites in Jilin Province. (**A**) Co-occurring genera analysis in six investigation sites; 23 genera co-occur in six investigation sites; site B2 has the fewest endemic genera and C2 has the most. (**B**) Co-occurring species analysis in six investigation sites; 11 species occur in all six investigation sites; site A1 has the most endemic species, while site B2 has the fewest. A1: Quanshuidong Forest Farm; A2: Lushuihe National Forest Park; B1: Shengli River Forest Farm; B2: Longwan National Forest Park; C1: Zuojia Region; C2: Jingyuetan National Forest Park.

A total of 691 species of macrofungi, belonging to 258 genera and 81 families, were identified (Table A1). There were 23 genera—including *Ampulloclitocybe* Redhead, Lutzoni, Moncalvo and Vilgalys, *Cortinarius*, and *Pleurotus*—identified at all six investigation sites (Figure 5A).

Furthermore, 11 species, such as *Ampulloclitocybe clavipes* (Pers.) Redhead, Lutzoni, Moncalvo and Vilgalys, *Daldinia concentrica* (Bolton) Ces. and De Not., and *Ganoderma applanatum* (Pers.) Pat., co-occurred at each survey site (Figure 5B). Site A1 has the most endemic species, while site B2 has the fewest.

### 3.3. Macrofungi Composition Types Analysis

**Figure 6:**
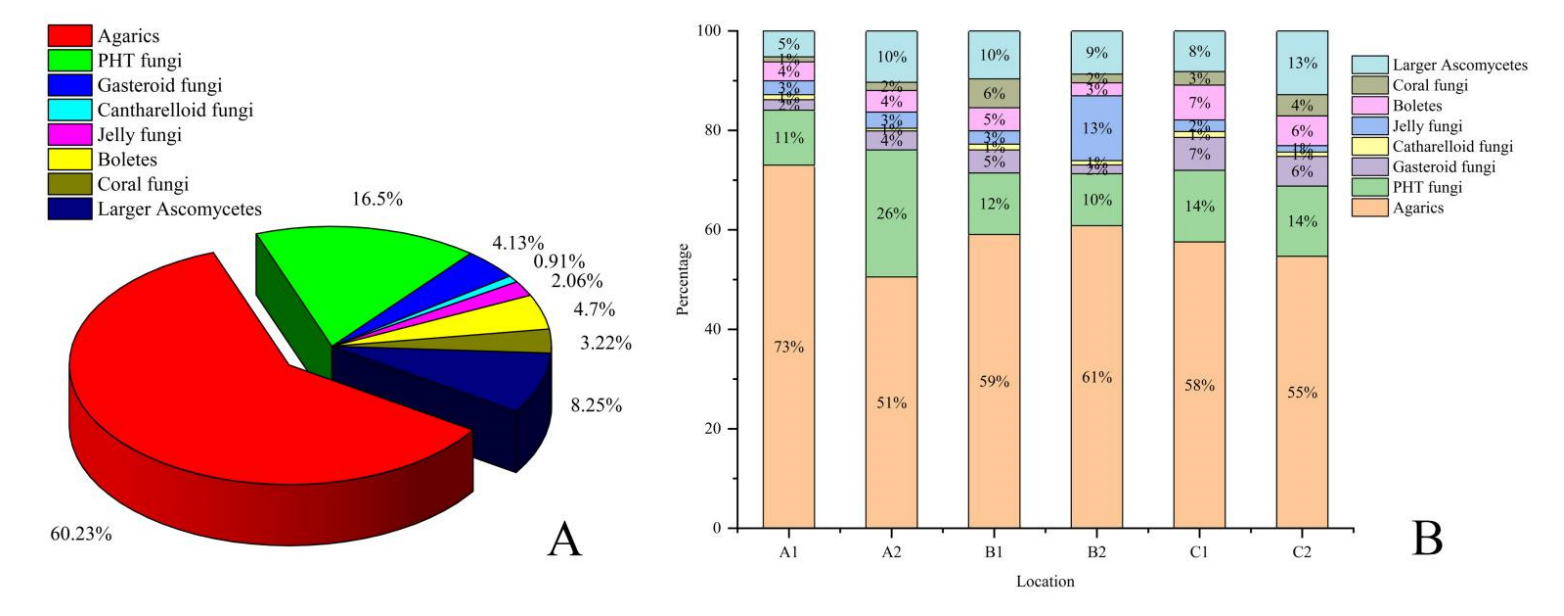
Distribution proportions for different types of macrofungi in six different investigation sites from Jilin Province. (**A**) Analysis of all recorded species composition types of our investigation sites; Agarics were the most common macrofungi while Canthralloid fungi were rare. (**B**) Species composition type analysis of every investigation site; Agarics were predominant at every site, Cantharelloid fungi were rare at all sites, and the compositions of macrofungi at six investigation sites were enormously different. PHT fungi: Polyporoid, Hyonaceous and Thelephoroid fungi; A1: Quanshuidong Forest Farm; A2: Lushuihe National Forest Park; B1: Shengli River Forest Farm; B2: Longwan National Forest Park; C1: Zuojia Region; C2: Jingyuetan National Forest Park.

According to the *Atlas of Chinese Macrofungal Resources* [37], the macrofungi species were divided into eight statistical categories. Agarics were the most common, accounting for 60.23% of the total, followed by PHT fungi, accounting for 16.50%. In contrast, Jelly fungi and Cantharelloid fungi were rarely reported, accounting for 2.06% and 0.91%, respectively (Figure 6A).

The statistical analysis of each investigation site (Figure 6B) showed that Agarics and PHT fungi were predominant at sites A1, A2, B1, B2, C1, and C2, while Agarics and Jelly fungi were the most common at site B2. Coral fungi and Cantharelloid fungi were rarely reported at sites A1, A2, and B2. Jelly fungi and Cantharelloid fungi were seldom reported at sites B1, C1, and C2. In summary, Cantharelloid fungi were rare in all investigations, while the compositions of macrofungi at six investigation sites were vastly different.

### 3.4. Ecological Characteristics of Macrofungi

According to the reference [6] and FUNGuild [47], the numbers of mycorrhizal macrofungi and saprophytic macrofungi were counted. The proportion of mycorrhizal macrofungi at site A1 was the highest (0.47), indicating that the forest structure at this site was the healthiest and the most stable. While the proportion was the lowest at site C1 (Table 3).

### 3.5. Analysis of α Diversity

**Figure 7:**
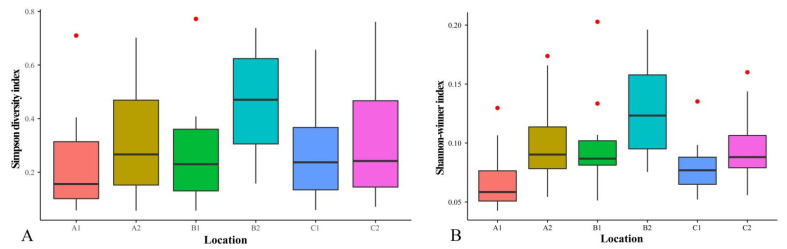
Diversity index analysis at genus level in six different investigation sites from Jilin Province. (**A**) Simpson diversity analysis in six different investigation sites from Jilin Province; the Simpson diversity index revealed site B2 was higher than the other five sites. (**B**) Shannon–Wiener diversity analysis in six different investigation sites from Jilin Province; the Shannon–Wiener diversity analysis showed site B2 was higher than the other five investigation sites. This result indicated that site B2 had the richest species diversity at the genus level and the most uniform distribution of species quantity. A1: Quanshuidong Forest Farm; A2: Lushuihe National Forest Park; B1: Shengli River Forest Farm; B2: Longwan National Forest Park; C1: Zuojia Region; C2: Jingyuetan National Forest Park.

The α diversity at six investigation sites was analyzed (Figure 7). The summary statistics from the Simpson diversity index for site B2 were significantly higher than those from the other five investigation sites. This result indicated that site B2 had the richest species diversity at the genus level and the most uniform distribution of species quantity (Figure 7A). The Shannon–Wiener index results also indicated that the diversity at site B2 was the highest, and the species were the richest (Figure 6B).

### 3.6. Analysis of Dominant Families (Genera)

**Figure 8:**
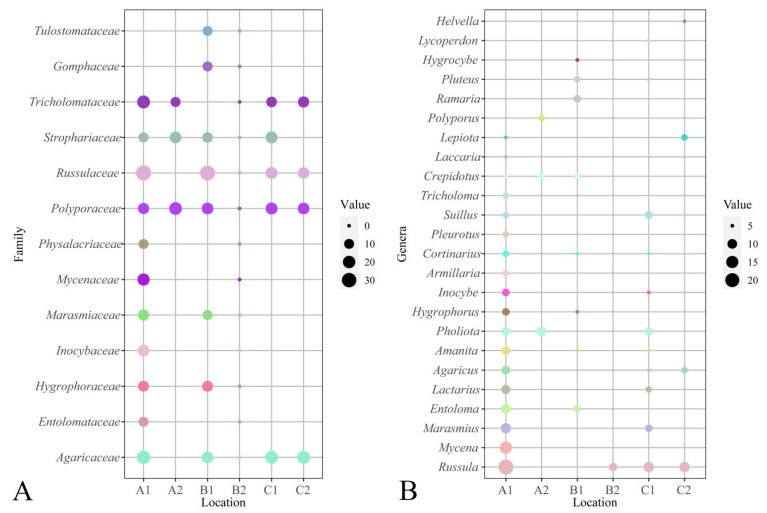
Dominant families and genera of six investigation sites from Jilin Province. (**A**) Dominant families (number of species more than ten of the family) analysis of six investigation sites from Jilin Province. (**B**) Dominant genera (number of species more than five of the genera) analysis of six investigation sites from Jilin Province. The results show site A1 contains more dominant families and genera in six investigation sites; in contrast, site B2 includes few. A1: Quanshuidong Forest Farm; A2: Lushuihe National Forest Park; B1: Shengli River Forest Farm; B2: Longwan National Forest Park; C1: Zuojia Region; C2: Jingyuetan National Forest Park.

According to the identification results, 81 families were recorded. The top ten families were *Russulaceae*, *Tricholomataceae*, *Agaricaceae*, etc., successively, containing 43.54% of the total species (Table 4). There were 24 families with only one species, accounting for 29.63%. The dominant families at each investigation site are shown in Figure 8A.

A total of 258 genera were reported in this study, among which the top ten genera were *Mycena* (Pers.) Roussel, *Cortinarius* (Pers.) Gray, *Lactarius* Pers., etc., accounting for 22.46% (Table 4). There were 137 genera with only one species, accounting for 53.10%. The dominant genera are shown in Figure 8B.

### 3.7. Relationships between Macrofungi and Environmental Factors

**Figure 9:**
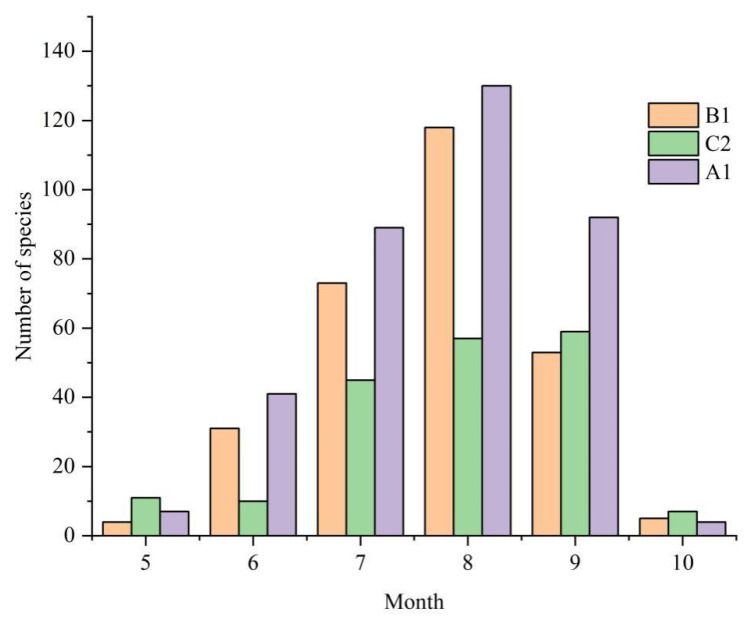
Relationship between macrofungi occurrence and month in three different investigation sites from Jilin Province. The results showed that they mostly arose from July to September. A1: Quanshuidong Forest Farm; B1: Shengli River Forest Farm; C2: Jingyuetan National Forest Park.

At first, the number of macrofungi collected from May to October at the three investigation sites was analyzed statistically. The results showed that they mainly arose from July to September, with minimal presence in May, June, and October (Figure 9).

**Figure 10:**
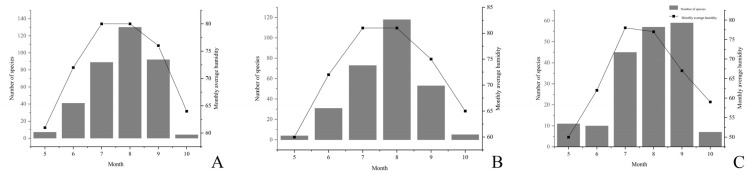
Effect of air humidity on the occurrence of macrofungi in three different investigation sites from Jilin Province. (**A**) Quanshuidong Forest Farm; (**B**) Shengli River Forest Farm; (**C**) Jingyuetan National Forest Park.

**Figure 11:**
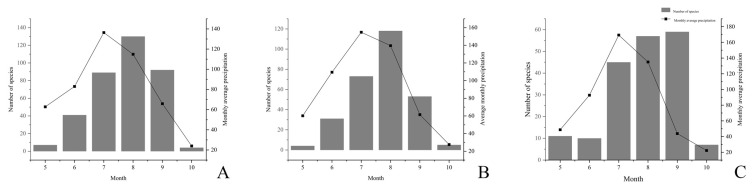
Effect of precipitation on the occurrence of macrofungi in three different investigation sites from Jilin Province. (**A**) Quanshuidong Forest Farm; (**B**) Shengli River Forest Farm; (**C**) Jingyuetan National Forest Park.

Secondly, the relationship between macrofungi and environmental facts—air humidity, precipitation, and temperature were also analyzed. Macrofungi occurrence was positively correlated with air humidity (Figure 10). When air humidity was higher, larger numbers of macrofungi were shown from July to September. Precipitation from May to October was positively correlated with macrofungi occurrence with a lag period (Figure 11).

**Figure 12:**
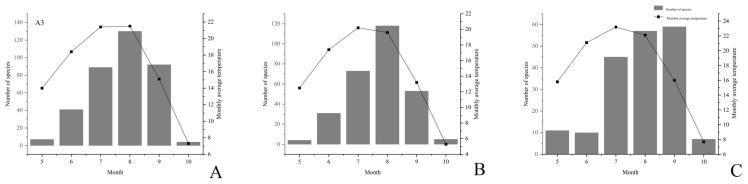
Effect of temperature on the occurrence of macrofungi in three different investigation sites from Jilin Province. (**A**) Quanshuidong Forest Farm; (**B**) Shengli River Forest Farm; (**C**) Jingyuetan National Forest Park.

Then, the relationship between the average temperature from May to October and macrofungi occurrence was also analyzed. The results showed that macrofungi occurrence at sites A1 (Figure 12A) and B2 (Figure 12B) was positively correlated with air temperature, and there was a relative lag period. However, numerous macrofungi occurred in September at site C2 (Figure 12C), while the monthly average temperature was significantly lower than from June to July. Further analysis of the meteorological data shows that the daily temperature difference at site C2 was significant in September, stimulating macrofungi formation.

**Figure 13:**
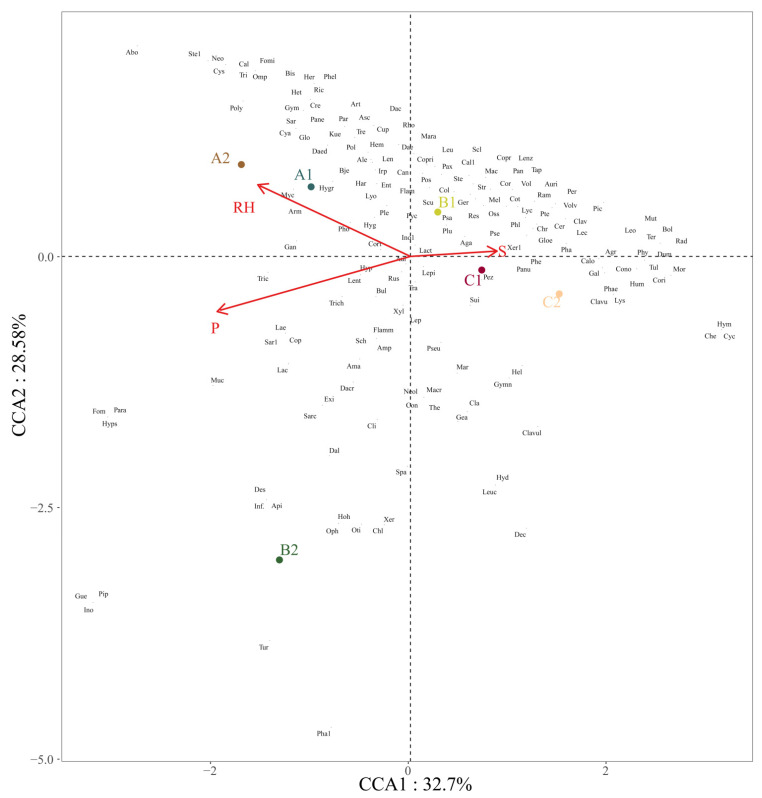
Canonical correspondence analysis (CCA) of the selected environmental factors and the recorded macrofungi species. All displayed environmental factors passed the most significant test (*p* < 0.05); P: mean precipitation from July to September; S: mean wind speed from July to September; RH: mean humidity from July to September; A1: Quanshuidong Forest Farm; A2: Lushuihe National Forest Park; B1: Shengli River Forest Farm; B2: Longwan National Forest Park; C1: Zuojia Region; C2: Jingyuetan National Forest Park. Letters are composed of the first three- or four-letter abbreviations of the scientific name, and the corresponding words are provided in Table A2.

At last, a canonical correspondence analysis (CCA) was performed on the genera with the top 50% species numbers recorded at the six investigation sites. Five environmental factors—adequate accumulated temperature (AT), monthly mean air temperature from (T), mean humidity (RH), mean precipitation (P), and mean wind speed from July to September (S)—were selected for CCA. The results (Figure 13) showed that all samples were roughly separated into six groups according to their corresponding locations. Eigenvalue axis 1 is higher than axis 2, with cumulative contributions of 32.70% and 28.50%, respectively. The selected environmental factors were found to impact the macrofungi occurrence. Of all the established ecological factors, the mean humidity from July to September, mean precipitation from July to September, and mean wind speed from July to September were the most significant factors.

### 3.8. Analysis of Flora Diversity

The six investigation sites were divided into three groups: Mt. Changbai area (A), containing Quanshuidong Forest Farm (A1) and Lushuihe National Forest Park (A2); Mt. Changbai Branch (B), comprising the Mt. Longgang Branch (Longwan National Forest Park, B1) and the Mt. Laoyeling Branch (Shengli Forest Farm, B2); and plain low hilly areas (C), encompassing the Zuojia Region (C1) and Jingyuetan National Forest Park (C2). The macrofungi composition was found to change when the mountainous region transited to the plains and low hills, and this was determined by calculating the similarity coefficient (s). The similarity decreased from 42.06% to 39.95% (Table 5).

Simultaneously, the macrofungi compositions of Mt. Changbai, its Laoyeling Branch (B1), and the Mt. Longgang Branch (B2) were compared. The similarity between Mt. Changbai and Laoyeling Branch (B1) was 37.23%, higher than the Mt. Longgang Branch.

**Figure 14:**
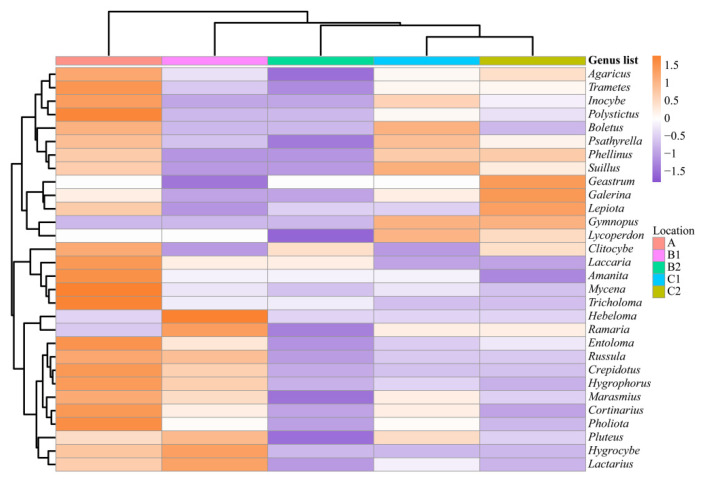
Complex heatmap of the macrofungi composition (genus level) at six investigation sites from Jilin Province. A1: Quanshuidong Forest Farm; A2: Lushuihe National Forest Park; B1: Shengli River Forest Farm; B2: Longwan National Forest Park; C1: Zuojia Region; C2: Jingyuetan National Forest Park.

The top 30 genera were selected to analyze the speciation differences (Figure 14). The composition of site C1 was the most similar to site C2, followed by site B2 and site B1, and site A was the least similar, which was consistent with the results for the similarity coefficient. The similarity of the species composition in Mt. Changbai Branch was lower than that in the plain low hilly area.

Substantial differences were seen in forming distinct genera among the six sites. The number of species in each genus was generally higher for area A and lower for site B2.

## 4. Discussion

### 4.1. The Influence of Environmental Factors on Macrofungi Occurrence

CCA at the genus level of recorded macrofungi at six investigation sites showed that the mean humidity, mean precipitation, and mean wind speed from July to September were the most significant environmental factors influencing the occurrence and distribution of macrofungi.

The effect of wind speed on macrofungi is integrated and multifaceted. The most direct impact is an expansion of the dispersal of the spores range, promoting species dispersal and affecting the macrofungi’s community structure by promoting the formation of dominant populations and reducing the macrofungi species richness within the same plant community [48]. Wind speed will also affect the oxygen content of the plant–macrofungi community. The high oxygen content will influence the oxygen content of soil [49], thus promoting hyphae respiration—the more energy released, the more promotion of mycelium growth [50]. Oxygen content will also affect the fruiting body morphogenesis, and elevated carbon dioxide will result in the formation of deformed mushrooms, thus affecting the macrofungi growth (e.g., the height of fruiting bodies lower than average) [51,52]. Wind speed will also affect soil moisture and air humidity [53]. From a positive perspective, water evaporation and transpiration will increase air humidity and adjust soil and air temperature, which benefits fruiting body formation. However, if the soil moisture evaporates excessively during spore germination and vegetative hyphal growth, excessive evaporation of the soil moisture will inhibit spore germination and promote hyphal reproductive growth or dormancy [54,55,56]. Furthermore, soil dryness caused by high winds may be a reason that macrofungi become gasteroid.

Precipitation will increase the soil water content, enabling resting spores to obtain sufficient water levels. For spores with thick walls, water immersion is essential. Sufficient soaking softens the walls, triggering enzymes hydrolysis of the spore’s peptidoglycan cortex, enabling the mycelium to germinate more efficiently [57]. Water immersion will also dissolve the substances that inhibit spore germination into the water and release dormancy [58]. Furthermore, water can promote spore respiration and sugar decomposition, provide energy for growth activities, and stimulate spores to secrete various enzymes to destroy cell wall structures [59]. With the gradual temperature increase, the spores were found to absorb enough water to germinate gradually. The suitable temperature and humidity conditions were sufficient for the mycelium to grow in large quantities, laying the foundations for macrofungi occurrence [60,61]. However, this phenomenon depends on vital mechanisms of the spore, for dead spores do not swell, and absorption varies with the viability of the spore [62]. The swelling of spores is usually more than twice its original size [63], and with further germination, the protoplasm volume can sometimes increase more than ten times.

Relative humidity mainly affects the dispersal of spores. If the air humidity is too high, the weight/volume will also increase, thus reducing the dispersal range of spores [64]. The evidence shows that the RH had no direct influence on the growth of macrofungi [53]. If water is available on the surface, macrofungi may grow at deficient air humidity levels [65,66]. RH may also influence the growth of mycelia. Excessively high RH would slow down or inhibit mycelium growth [67].

Mushrooms also arise from primordia that their formation and differentiation are influenced by environmental factors such as precipitation and temperature. From 1993 to 2007, Krebs et al. [68] found that mushroom production could be predicted by summer rainfall, in Yukon, the mushroom production is positively correlated with precipitation. Low humidity will slow down the growth rate during primordia formation [69]. The temperature is also another critical factor. The formation of some mushroom’s primordia requires low-temperature stimulation, such as *Flammlina filiformis* (Z.W. Ge, X.B. Liu, and Zhu L. Yang) P.M. Wang, Y.C. Dai, E. Horak, and Zhu L. Yang. The diverse climate types and environments allow different macrofungi to specialize and thrive [70].

### 4.2. The Influence of Vegetation on Macrofungi Occurrence

The Mt. Longgang and Mt. Laoyeling branches both belong to Mt. Changbai. However, the species richness in the Longwan National Forest Park (B2) was found to be higher than that at Mt. Changbai (A) and its Mt. Laoyeling branch (B1). This phenomenon may be due to the differences in their vegetation [71]. Mt. Changbai and its Mt. Laoyeling Branch are mainly covered by coniferous trees, including *Pinus* spp., *Picea* spp., etc. In contrast, Longgang Branch (Longwan National Forest Park, B2) is primarily covered with broadleaf mixed forests, such as *Quercus mongolica* and some pine forests. Macrofungi can show preferences for broadleaf or coniferous trees, vegetation, or substrate specificity might have contributed to the evolution of macrofungi [11,70]. Our result (Figure 6) shows that the typical composition of recorded macrofungi varied in proportion across the six investigation sites. Jelly fungi, for example, at site B2 reached 13%; however, they were only 1–3% at the other investigation sites. Furthermore, deadwood fungi prefer different deadwood characteristics (host species, decay, etc.), and thus, species composition changes can occur about these characteristics [72]. It is evidenced that macrofungi species are usually more abundant in broadleaf forests than in coniferous forests [11]. According to our calculations, the wood and litter saprotroph macrofungi reached 51.7% at site B2, while sites A1, A2, and B1 were 50.3%, 47.9%, and 46.3%, respectively. In addition, plant community composition determines understory light availability, humidity, and litter composition [73]. At the same time, many macrofungal species have host associations with particular plant species; for example, Tuo et al. revealed that the quantity of EM fungi in Wunvfeng National Forest Park, China, was positively correlated with the amount of *Q. mongolica* [6]. Based on our results, site A1 had the highest proportion of EM fungi at 45.78% and site B2 had the lowest at 28.57% among Mt. Changbai and its branch sites. The balance between mycorrhizal macrofungi and saprophytic macrofungi is a reference to forest conditions [74,75,76,77]. In a healthy forest, the number of mycorrhizal macrofungi often exceeds the number of saprophytic macrofungi [78,79]. Moreover, the plant community constitutes an abiotic factor of crucial importance for fungal composition [80,81]. However, some studies have demonstrated that the contribution of plant communities to the impact of macrofungi communities is only 1–10% [82]. Therefore, the effects of plant communities on macrofungi require further investigation.

### 4.3. The Influence of Topography on Macrofungi Occurrence

The ratio of mycorrhizal macrofungi to saprophytic macrofungi decreased with the transition from the eastern mountains to the central plains. Unlike light or soil properties, the topography is an indirect environmental variable [83,84]. Topography is considered an essential driver of micro-habitat diversity in forest ecosystems [85,86], as different topographies result in various micro-habitats. Different micro-habitats can favor the occurrence of a wider variety of macrofungal species [84], thus leading to different macrofungi compositions, which we observed in our results. In our findings, the proportion of macrofungal composition types varied across six survey sites (Figure 6). Moreover, the species numbers for each genus shifted with topography (Figure 14). For example, the *Lepiota* and *Geastrum* species were most common at site C2; however, they were considered rare at the other sites.

Different macrofungi compositions eventually result in variations in species similarity. Species similarity decreased with the transition from the mountainous area to the plains area in this investigation. Furthermore, the similarity between Mt. Changbai and its Laoyeling Branch (Shengli Forestry Farm, B1) was higher than between Mt. Changbai and the Mt. Longgang branch (Longwan National Forest Park, B2). Based on the comparison of the representative vegetation and soil types of the three areas, the representative forest types and soil types in the Laoyeling Branch and Mt. Changbai area were highly similar, and it is speculated that the occurrence of macrofungi is not only related to vegetation but also closely related to soil types. Soil type influenced spore density and the percentage of mycorrhizal colonization of roots, where high spore density was not necessarily connected with intensive mycorrhizal development [87].

## 5. Conclusions

The occurrence of macrofungi is closely related to vegetation. By comparing sites B1 and B2, we found that the macrofungal abundance increased with increasing proportions of broadleaf trees, and specific genera were present at every survey site. Moreover, the nutritional patterns of co-occurring genera (species) were analyzed, most of which were saprophytic macrofungi.

The mycorrhizal:saprophytic ratios decreased with the transition from mountains to plains. The mycorrhizal:saprophytic ratios were consistently higher in the northeast than the southwest sites in the Mt. Changbai region and its branches.

Species similarity decreased with the transition from the mountainous area to the plains area; in addition, the species similarity between the Laoyeling Branch (B1) and Mt. Changbai (A) is higher than that between the Mt. Longgang Branch (B2) and Mt. Changbai (A).

The main environmental factors affecting macrofungi occurrence from the eastern mountains to the central plains of Jilin Province are the air humidity (RH), precipitation (P), and wind speed (S) from July to September. Our canonical correspondence analysis reveals the importance of wind speed in macrofungal occurrence.

## Figures and Tables

**Table 1:** Geographical coordinates and vegetation of the investigation sites.

Investigation Sites	Longitude	Latitude	Vegetation	Soil Type	Investigation Time
Quanshuidong Forest Farm (A1)	128.89° E	42.68° N	*Pinus* and broadleaf mixed forest, *Quercus* and *Poplar* forest, *coniferous* forests (*Pinus koraiensis*), *Taxus cuspidata*, *Salix* spp., etc.	Dark brunisolic soil and planosol soil	2014–2016
Lushuihe National Forest Park (A2)	128.01° E	42.55° N			2013–2015
Mt. Laoyeling (Shengli River Forest Farm, B1)	127.83° E	43.69° N	*Pinus koraiensis* and broadleaf mixed forest, larch forest, *Quercus mongolica* forest, *Acer truncatum*, *Tilia amurensis*, and *Fraxinus mandshurica*, etc.	Dark brunisolic soil and planosol soil	2005–2007
Mt. Longgang (Longwan National Forest Park, B2)	126.45° E	42.37° N	*Quercus mongolica* forest and broadleaf mixed forest	Dark brunisolic soil	2011–2013
Zuojia Region (C1)	126.16° E	44.03° N	*Quercus mongolica* forest, spruce forest, and pine forest	Black soil and paddy soil	2011–2013
Jingyuetan national forest park (C2)	125.48° E	43.79° N	Larch forest, *Pinus* forest, and *Quercus mongolica* forest	Dark brunisolic soil	2003–2006

**Table 2:** Meteorological conditions and types of soil at the investigation sites.

Investigation Site	The Average Temperature from July to September/°C (T)	The Average Relative Humidity from July to September/% (RH)	The Average Monthly Precipitation from July to September/mm (P)	The Average Wind Speed from July to September/m/s (S)	The Accumulated Temperature from July to September/°C (AT)
Quanshuidong Forest Farm (A1)	18.60	80.67	152.27	1.57	1715.90
Lushuihe Town (A2)	19.60	81.67	143.27	1.67	1698.9
Mt. Laoyeling—Shengli River Forest Farm (B1)	17.67	79.00	118.63	1.90	1629.80
Mt. Longgang—Longwan National Forest Park (B2)	19.53	78.33	170.77	1.23	1746.40
Zuojia Region (C1)	19.93	77.00	124.43	2.03	1838.60
Jingyuetan National Forest Park (C2)	20.43	74.00	116.03	2.83	1884.30

**Table 3:** Mycorrhizal:saprophytic macrofungi ratios of the investigation sites from Jilin Province.

Sites	A1	A2	B1	B2	C1	C2
Mycorrhizal	92	76	77	30	60	57
Saprophytic	197	170	181	75	196	175
Ratio/%	0.47	0.45	0.43	0.40	0.31	0.33

**Table 4:** Top 10 families and genera in six different investigation sites from Jilin Province.

No.	Family	Numbers of Species	Percentage	Genus	Numbers of Species	Percentage
1	*Agaricaceae*	52	7.07%	*Lactarius*	20	2.72%
2	*Polyporaceae*	50	6.80%	*Mycena*	19	2.59%
3	*Tricholomataceae*	41	5.58%	*Cortinarius*	18	2.45%
4	*Inocybaceae*	34	4.63%	*Marasmius*	18	2.45%
5	*Strophariaceae*	33	4.49%	*Pholiota*	18	2.45%
6	*Hygrophoraceae*	25	3.40%	*Agaricus*	16	2.18%
7	*Marasmiaceae*	25	3.40%	*Entoloma*	16	2.18%
8	*Mycenaceae*	23	3.13%	*Amanita*	14	1.90%
9	*Cortinariaceae*	19	2.59%	*Crepidotus*	13	1.77%
10	*Omphalotaceae*	18	2.45%	*Inocybe*	13	1.77%

**Table 5:** Similarity comparison between Mt. Changbai, its branches, and five other investigation sites.

Location	B	C	B1	B2	C1	C2
S/%	42.06	39.95	37.23	32.39	26.85	30.88

## Data Availability

Not applicable.

## Appendix A

**Table A1:** Macrofungi list of collected species in three different landforms from Jilin Province.

Scientific Name	Distribution						Nutritional Mode	Economic Value	Categories	GenBank Accession Number
	A1	A2	B1	B2	C1	C2				
*Abortiporus biennis* (Bull.) Singer	√						SS	Medicinal	PHT fungi	
*Agaricus abruptibulbus* Peck	√						SS	Edible, Poisonous	Agarics	ON683434
*Agaricus arvensis* Schaeff.					√		SS	Edible, Medicinal	Agarics	
*Agaricus bresadolanus* Bohus	√						SS	Edible, Medicinal	Agarics	
*Agaricus campestris* L.	√		√		√	√	SS	Edible, Medicinal	Agarics	
*Agaricus comtulus* Fr.	√			√			SS	Edible	Agarics	
*Agaricus micromegethus* Peck	√				√	√	SS	Edible, Medicinal	Agarics	
*Agaricus moelleri* Wasser			√			√	SS	Edible, Poisonous	Agarics	ON683435
*Agaricus perrarus* Fr.						√	SS	Others	Agarics	
*Agaricus placomyces* Peck					√		SS	Edible, Medicinal	Agarics	
*Agaricus purpurellus* F.H. Møller						√	SS	Others	Agarics	
*Agaricus silvaticus* Schaeff.			√	√			SS	Edible, Medicinal	Agarics	
*Agaricus silvicolae-similis* Bohus and Locsmándi	√				√		SS	Others	Agarics	
*Agaricus subrufescens* Peck	√				√		SS	Edible, Medicinal	Agarics	
*Agaricus subrutilescens* (Kauffman) Hotson and D.E. Stuntz	√		√			√	SS	Edible, Medicinal	Agarics	
*Agaricus sylvaticus* Schaeff.	√					√	SS	Edible, Medicinal	Agarics	
*Agrocybe pediades* (Fr.) Fayod					√	√	SS	Edible, Medicinal	Agarics	
*Agrocybe praecox* (Pers.) Fayod	√		√		√	√	SS	Edible, Medicinal	Agarics	ON683416
*Aleuria aurantia* (Pers.) Fuckel	√				√		SS	Edible, Medicinal, Poisonous	Larger Ascomycetes	
*Aleurodiscus stereoides* Yasuda						√	SS	Others	PHT fungi	
*Amanita ceciliae* (Berk. and Broome) Bas	√			√			EM	Others	Agarics	ON683421
*Amanita fulva* Fr.					√	√	EM	Others	Agarics	
*Amanita hemibapha* (Berk. and Broome) Sacc.			√		√		EM	Edible, Medicinal	Agarics	
*Amanita imazekii* T. Oda, C. Tanaka and Tsuda	√			√			EM	Edible	Agarics	
*Amanita longistriata* S. Imai	√		√		√		EM	Poisonous	Agarics	
*Amanita nivalis* Grev.	√			√			EM	Edible	Agarics	
*Amanita pantherina* (DC.) Krombh	√		√	√	√		EM	Poisonous	Agarics	
*Amanita phalloides* (Vaill. ex Fr.) Link	√					√	EM	Others	Agarics	ON683436
*Amanita porphyria* Alb. and Schwein.			√				EM	Others	Agarics	ON683437
*Amanita spreta* (Peck) Sacc.					√		EM	Others	Agarics	
*Amanita subjunquillea* S. Imai	√		√	√		√	EM	Poisonous	Agarics	ON683438
*Amanita vaginata* (Bull.) Lam.	√		√	√	√	√	EM	Edible, Medicinal, Poisonous	Agarics	ON683439
*Amanita virosa* Secr.	√			√			EM	Medicinal, Poisonous	Agarics	
*Amanitopsis fulva* (Fr.) W.G. Sm.					√		EM	Others	Agarics	
*Ampulloclitocybe clavipes* (Pers.) Redhead, Lutzoni, Moncalvo and Vilgalys	√	√	√	√	√	√	LS	Edible, Medicinal, Poisonous	Agarics	
*Apioperdon pyriforme* (Schaeff.) Vizzini	√			√	√		EM	Edible, Medicinal	Gasteroid fungi	
*Armillaria borealis* Marxm. and Korhonen	√			√			WS	Edible, Medicinal	Agarics	
*Armillaria cepistipes* Velen.	√				√		WS	Others	Agarics	
*Armillaria gallica* Marxm. and Romagn.	√						WS	Edible, Medicinal	Agarics	ON683440
*Armillaria mellea* (Vahl) P. Kumm.	√	√		√	√		WS	Edible, Medicinal	Agarics	
*Armillaria ostoyae* (Romagn.) Herink	√	√					WS	Edible, Medicinal	Agarics	
*Armillaria sinapina* Bérubé and Dessur.	√						WS	Edible, Medicinal	Agarics	ON683422
*Armillariella mellea* (Vahl) P. Karst.		√	√		√	√	WS	Edible, Medicinal	Agarics	
*Artomyces pyxidatus* (Pers.) Jülich	√	√	√		√		WS	Edible	Coral fungi	
*Ascocoryne cylichnium* (Tul.) Korf		√	√		√		WS	Others	Larger Ascomycetes	
*Astraeus hygrometricus* (Pers.) Morgan	√						SS	Medicinal	Gasteroid fungi	
*Atheniella adonis* (Bull.) Redhead, Moncalvo, Vilgalys, Desjardin, and B.A. Perry	√			√			LS	Others	Agarics	
*Auricularia cornea* Ehrenb.		√	√	√	√	√	WS	Edible, Medicinal	Jelly fungi	
*Auricularia heimuer* F. Wu, B.K. Cui, and Y.C. Dai		√	√	√			WS	Edible, Medicinal	Jelly fungi	
*Auricularia mesenterica* (Dicks.) Pers.		√			√	√	WS	Others	Jelly fungi	
*Auricularia nigricans* (Sw.) Birkebak, Looney and Sánchez-García			√		√	√	WS	Edible, Medicinal	Jelly fungi	
*Auriscalpium vulgare* Gray			√		√	√	WS	Others	PHT fungi	
*Baeospora myriadophylla* (Peck) Singer		√					LS	Others	Agarics	
*Bisporella sulfurina* (Quél.) S.E. Carp.	√		√				WS	Others	Larger Ascomycetes	
*Bjerkandera adusta* (Willd.) P. Karst.	√				√		WS	Medicinal	PHT fungi	
*Bolbitius vitellinus* (Pers.) Fr.					√		SS	Others	Agarics	
*Bolbitius vitellinus* (Pers.) Fr.	√			√			EM	Others	Boletes	
*Boletinellus merulioides* (Schwein.) Murrill			√			√	EM	Others	Boletes	
*Boletus edulis* Bull.	√		√	√	√		EM	Edible	Boletes	
*Boletus subtomentosus* J.A. Palmer	√						EM	Others	Boletes	
*Boletus subvelutipes* Peck						√	EM	Others	Boletes	
*Boletus yunnanensis* W.F. Chiu					√		EM	Poisonous	Boletes	
*Bovista pusilla* (Batsch) Pers.	√						SS	Others	Gasteroid fungi	
*Bovista pusilliformis* (Kreisel) Kreisel						√	SS	Others	Gasteroid fungi	
*Bulgaria inquinans* (Pers.) Fr.		√					WS	Edible, Medicinal, Poisonous	Larger Ascomycetes	
*Byssomerulius corium* (Pers.) Parmasto						√	WS	Others	PHT fungi	
*Calocera cornea* (Batsch) Fr.	√	√					WS	Others	Jelly fungi	
*Calocera viscosa* (Pers.) Fr.			√				WS	Medicinal, Poisonous	Jelly fungi	
*Calocybe gambosa* (Fr.) Donk	√					√	SS	Edible, Medicinal	Agarics	
*Calocybe ionides* (Bull.) Donk						√	SS	Edible	Agarics	
*Calvatia craniiformis* (Schwein.) Fr. ex De Toni	√				√		SS	Edible, Medicinal	Gasteroid fungi	
*Calvatia lilacina* (Mont. and Berk.) Henn.					√		SS	Edible, Medicinal	Gasteroid fungi	
*Calvatia tatrensis* Hollós			√				SS	Medicinal	Gasteroid fungi	
*Camarophyllus pratensis* (Pers.) P. Kumm.					√		SS	Others	Agarics	
*Campanella tristis* (G. Stev.) Segedin	√	√					WS	Others	Agarics	
*Cantharellus cibarius* Fr.	√	√	√		√	√	EM	Edible, Medicinal	Cantharelloid fungi	ON683495
*Cantharellus minor* Peck	√				√		EM	Edible, Medicinal	Cantharelloid fungi	
*Cerioporus squamosus* (Huds.) Quél.	√		√		√	√	WS	Medicinal	PHT fungi	
*Cerioporus varius* (Pers.) Zmitr. and Kovalenko					√		WS	Edible	PHT fungi	
*Ceriporia tarda* (Berk.) Ginns	√			√			WS	Others	PHT fungi	
*Cheilymenia coprinaria* (Cooke) Boud.					√	√	WS	Others	Larger Ascomycetes	
*Chlorociboria aeruginascens* (Nyl.) Kanouse				√	√		WS	Others	Larger Ascomycetes	
*Chroogomphus purpurascens* (Lj.N. Vassiljeva) M.M. Nazarova		√		√			EM	Edible	Boletes	
*Chroogomphus roseolus* Y.C. Li and Zhu L. Yang					√		EM	Edible, Medicinal	Boletes	
*Chroogomphus rutilus* (Schaeff.) O.K. Mill.		√	√		√	√	EM	Edible, Medicinal	Boletes	ON683423
*Chroogomphus tomentosus* (Murrill) O.K. Mill.					√	√	EM	Others	Boletes	
*Chrysomphalina aurantiaca* (Peck) Redhead	√				√	√	WS	Others	Agarics	
*Clavaria fragilis* Holmsk.				√		√	SS	Medicinal, Poisonous	Coral fungi	
*Clavaria vermicularis* Sw.			√				SS	Edible	Coral fungi	
*Clavariadelphus pistillaris* (L.) Donk			√		√		LS	Edible, Poisonous	Coral fungi	ON683441
*Clavicorona pyxidata* (Pers.) Doty						√	WS	Others	Coral fungi	
*Clavulina coralloides* (L.) J. Schröt.	√				√		LS	Edible	Coral fungi	ON683424
*Clavulinopsis corniculata* (Schaeff.) Corner			√			√	LS	Edible	Coral fungi	
*Clavulinopsis fusiformis* (Sowerby) Corner			√	√			LS	Edible	Coral fungi	
*Clavulinopsis helvola* (Pers.) Corner						√	LS	Edible	Coral fungi	
*Clitocybe infundibuliformis* (Schaeff.) Quél.				√	√	√	LS	Edible, Medicinal	Agarics	
*Clitocybe nebularis* (Batsch) P. Kumm.		√					LS	Edible, Medicinal	Agarics	
*Clitocybe odora* (Bull.) P. Kumm.						√	LS	Edible, Medicinal	Agarics	ON683417
*Clitocybe phyllophila* (Pers.) P. Kumm.	√			√	√		LS	Poisonous	Agarics	
*Clitopilus prunulus* (Scop.) P. Kumm.	√	√		√			LS	Edible	Agarics	
*Collybia nivea* (Mont.) Dennis						√	SS	Others	Agarics	
*Collybiopsis confluens* (Pers.) R.H. Petersen		√	√		√	√	SS	Others	Agarics	ON683418
*Collybiopsis peronata* (Bolton) R.H. Petersen	√		√		√		SS	Others	Agarics	
*Coltricia cinnamomea* (Jacq.) Murrill		√	√		√	√	SS	Others	PHT fungi	
*Coltricia perennis* (L.) Murrill	√	√					SS	Others	PHT fungi	
*Connopus acervatus* (Fr.) K.W. Hughes, Mather and R.H. Petersen		√	√	√	√	√	SS	Edible	Agarics	
*Conocybe lactea* (J.E. Lange) Métrod			√		√	√	SS	Medicinal	Agarics	
*Conocybe tenera* (Schaeff.) Kühner					√		SS	Poisonous	Agarics	
*Coprinellus disseminatus* (Pers.) J.E. Lange	√		√	√	√	√	SS	Poisonous	Agarics	
*Coprinellus micaceus* (Bull.) Vilgalys, Hopple and Jacq. Johnson	√	√	√	√	√		SS	Medicinal, Poisonous	Agarics	
*Coprinopsis atramentaria* (Bull.) Redhead, Vilgalys and Moncalvo		√	√		√		SS	Edible, Medicinal, Poisonous	Agarics	
*Coprinopsis cinerea* (Schaeff.) Redhead, Vilgalys and Moncalvo					√	√	SS	Medicinal	Agarics	
*Coprinopsis insignis* (Peck) Redhead, Vilgalys & Moncalvo			√				SS	Medicinal	Agarics	
*Coprinopsis lagopus* (Fr.) Redhead, Vilgalys and Moncalvo	√	√	√				SS	Medicinal	Agarics	
*Coprinopsis picacea* (Bull.) Redhead, Vilgalys and Moncalvo					√		SS	Poisonous	Agarics	
*Coprinus comatus* (O.F. Müll.) Pers.		√	√			√	DS	Edible, Medicinal, Poisonous	Agarics	
*Coprinus micaceus* (Bull.) Fr.						√	DS	Medicinal, Poisonous	Agarics	
*Coprinus plicatilis* (Curtis) Fr.		√	√				DS	Others	Agarics	
*Coprinus sterquilinus* (Fr.) Fr.	√		√	√			DS	Medicinal	Agarics	
*Cordyceps farinosa* (Holmsk.) Kepler, B. Shrestha and Spatafora						√	EI	Others	Larger Ascomycetes	
*Cordyceps militaris* (L.) Fr.		√	√		√	√	EI	Edible, Medicinal	Larger Ascomycetes	
*Cordyceps nutans* Pat.		√					EI	Others	Larger Ascomycetes	
*Coriolopsis gallica* (Fr.) Ryvarden			√		√	√	WS	Others	PHT fungi	
*Cortinarius alboviolaceus* (Pers.) Fr.		√					EM	Edible	Agarics	
*Cortinarius armillatus* (Fr.) Fr.		√					EM	Others	Agarics	
*Cortinarius bovinus* Fr.					√		EM	Edible, Medicinal	Agarics	
*Cortinarius caerulescens* (Schaeff.) Fr.			√		√		EM	Others	Agarics	
*Cortinarius callochrous* (Pers.) Gray	√		√	√			EM	Edible, Poisonous	Agarics	
*Cortinarius caperatus* (Pers.) Fr.			√				EM	Edible, Medicinal	Agarics	
*Cortinarius cinnamomeus* (L.) Gray			√	√			EM	Edible, Medicinal, Poisonous	Agarics	
*Cortinarius collinitus* (Sowerby) Gray					√		EM	Edible, Medicinal	Agarics	
*Cortinarius colymbadinus* Fr.					√		EM	Others	Agarics	
*Cortinarius elatior* Fr.	√				√		EM	Others	Agarics	
*Cortinarius galeroides* Hongo	√		√				EM	Others	Agarics	
*Cortinarius lilacinus* Peck				√			EM	Poisonous	Agarics	
*Cortinarius longipes* Peck						√	EM	Others	Agarics	
*Cortinarius multiformis* Fr.	√						EM	Edible	Agarics	
*Cortinarius orellanus* Fr.	√						EM	Poisonous	Agarics	
*Cortinarius purpurascens* Fr.			√				EM	Edible	Agarics	ON683425
*Cortinarius sanguineus* (Wulfen) Gray			√			√	EM	Medicinal, Poisonous	Agarics	
*Cortinarius trivialis* J.E. Lange	√						EM	Edible, Poisonous	Agarics	
*Cotylidia diaphana* (Cooke) Lentz	√						LS	Others	PHT fungi	
*Cotylidia komabensis* (Henn.) D.A. Reid			√		√	√	LS	Others	PHT fungi	
*Craterellus undulatus* (Pers.) E. Campo and Papetti	√				√	√	EM	Edible	Agarics	
*Crepidotus applanatus* (Pers.) P. Kumm.		√	√				WS	Others	Agarics	
*Crepidotus badiofloccosus* S. Imai		√					WS	Others	Agarics	
*Crepidotus betulae* Murrill		√	√				WS	Others	Agarics	
*Crepidotus brasiliensis* Rick		√		√			WS	Medicinal	Agarics	
*Crepidotus epibryus* (Fr.) Quél.	√						WS	Others	Agarics	
*Crepidotus fulvotomentosus* (Peck) Peck			√				WS	Others	Agarics	
*Crepidotus herbarum* Sacc.			√				WS	Others	Agarics	
*Crepidotus mollis* (Schaeff.) Staude	√	√			√	√	WS	Others	Agarics	
*Crepidotus nephrode* (Berk. and M.A. Curtis) Sacc.			√				WS	Others	Agarics	
*Crepidotus nephrodes* (Berk. and M.A. Curtis) Sacc.	√						WS	Others	Agarics	
*Crepidotus palmularis* (Berk. and M.A. Curtis) Sacc.	√		√				WS	Others	Agarics	
*Crepidotus sulphurinus* Imazeki and Toki	√	√	√				WS	Others	Agarics	
*Crepidotus variabilis* (Pers.) P. Kumm.			√				WS	Others	Agarics	
*Crinipellis stipitaria* (Fr.) Pat.					√		LS	Others	Agarics	
*Cuphophyllus pratensis* (Pers.) Bon		√	√				SS	Edible	Agarics	ON683442
*Cuphophyllus virgineus* (Wulfen) Kovalenko	√		√		√		SS	Others	Agarics	
*Cyanosporus caesius* (Schrad.) McGinty		√	√		√	√	WS	Others	PHT fungi	
*Cyathus stercoreus* (Schwein.) De Toni					√		WS	Medicinal	Gasteroid fungi	
*Cyathus striatus* Willd.		√					WS	Medicinal	Gasteroid fungi	
*Cyclocybe cylindracea* (DC.) Vizzini and Angelini					√		SS	Edible, Medicinal	Agarics	
*Cyclocybe erebia* (Fr.) Vizzini and Matheny						√	SS	Edible, Medicinal	Agarics	
*Cystoderma amianthinum* (Scop.) Fayod	√	√	√				SS	Edible	Agarics	
*Cystoderma fallax* A.H. Sm. and Singer		√					SS	Edible	Agarics	
*Cystodermella granulosa* (Batsch) Harmaja		√					SS	Edible	Agarics	
*Dacrymyces chrysospermus* Berk. and M.A. Curtis	√	√				√	WS	Others	Jelly fungi	
*Dacrymyces palmatus* Bres.			√				WS	Medicinal	Jelly fungi	ON683443
*Dacryopinax spathularia* (Schwein.) G.W. Martin	√	√		√	√	√	WS	Others	Jelly fungi	
*Daedalea dickinsii* Yasuda		√	√		√		WS	Medicinal	PHT fungi	ON683444
*Daedaleopsis confragosa* (Bolton) J. Schröt.		√			√		WS	Others	PHT fungi	
*Daedaleopsis tricolor* (Bull.) Bondartsev and Singer	√	√			√		WS	Medicinal	PHT fungi	ON683426
*Daldinia concentrica* (Bolton) Ces. and De Not.	√	√	√	√	√	√	WS	Medicinal, Poisonous	Larger Ascomycetes	
*Daldinia grandis* Child				√			WS	Others	Larger Ascomycetes	
*Deconica coprophila* (Bull.) P. Karst.					√	√	DS	Poisonous	Agarics	
*Deconica merdaria* (Fr.) Noordel.					√		DS	Poisonous	Agarics	
*Desarmillaria tabescens* (Scop.) R.A. Koch and Aime	√	√	√				SS	Edible, Medicinal, Poisonous	Agarics	
*Descolea flavoannulata* (Lj.N. Vassiljeva) E. Horak				√	√		SS	Edible	Agarics	
*Dumontinia tuberosa* (Bull.) L.M. Kohn			√		√	√	SS	Others	Larger Ascomycetes	
*Entoloma abortivum* (Berk. and M.A. Curtis) Donk		√					SS	Edible, Medicinal	Agarics	
*Entoloma albipes* Hesler	√				√		SS	Others	Agarics	
*Entoloma chamaecyparidis* (Hongo) Hongo						√	SS	Others	Agarics	
*Entoloma clypeatum* (L.) P. Kumm.	√	√	√		√	√	SS	Medicinal, Poisonous	Agarics	
*Entoloma japonicum* (Hongo) Hongo and Izawa						√	SS	Others	Agarics	
*Entoloma lividum* Quél.			√				SS	Others	Agarics	
*Entoloma murinipes* (Murrill) Hesler	√			√			SS	Others	Agarics	
*Entoloma murrillii* Hesler	√						SS	Others	Agarics	
*Entoloma politum* (Pers.) Noordel.	√			√			SS	Others	Agarics	
*Entoloma rhodopolium* (Fr.) P. Kumm.	√		√	√	√	√	SS	Medicinal, Poisonous	Agarics	
*Entoloma sinuatum* (Bull.) P. Kumm.	√	√			√		SS	Medicinal, Poisonous	Agarics	
*Entoloma speculum* (Fr.) Quél.	√		√	√			SS	Others	Agarics	
*Entoloma umbilicatum* Dennis	√		√				SS	Others	Agarics	
*Entonaema liquescens* Möller	√	√		√			WS	Others	Larger Ascomycetes	
*Exidia glandulosa* (Bull.) Fr.	√		√	√	√		WS	Edible, Poisonous	Jelly fungi	
*Flammulaster erinaceellus* (Peck) Watling	√	√			√	√	WS	Others	Agarics	
*Flammulina filiformis* (Z.W. Ge, X.B. Liu, and Zhu L. Yang) P.M. Wang, Y.C. Dai, E. Horak, and Zhu L. Yang	√	√	√	√	√	√	WS	Edible	Agarics	
*Fomes fomentarius* (L.) Fr.	√	√		√			WS	Medicinal	PHT fungi	ON683445
*Fomitopsis betulina* (Bull.) B.K. Cui, M.L. Han, and Y.C. Dai			√				WS	Medicinal	PHT fungi	
*Fomitopsis officinalis* (Vill.) Bondartsev and Singer		√	√				WS	Medicinal	PHT fungi	
*Fomitopsis pinicola* (Sw.) P. Karst.		√					WS	Medicinal	PHT fungi	ON683446
*Galerina helvoliceps* (Berk. and M.A. Curtis) Singer						√	WS	Poisonous	PHT fungi	
*Galerina marginata* (Batsch) Kühner		√				√	SS	Poisonous	PHT fungi	
*Galerina vittaeformis* (Fr.) Singer					√		SS	Others	Agarics	
*Galiella amurensis* (Lj.N. Vassiljeva) Raitv.		√					SS	Others	Larger Ascomycetes	
*Ganoderma applanatum* (Pers.) Pat.	√	√	√	√	√	√	WS	Medicinal	PHT fungi	
*Ganoderma tsugae* Murrill	√	√			√		WS	Medicinal	PHT fungi	
*Geastrum fimbriatum* Fr.		√		√			LS	Medicinal	Gasteroid fungi	
*Geastrum pectinatum* Pers.						√	LS	Others	Gasteroid fungi	
*Geastrum saccatum* Fr.			√	√	√	√	LS	Medicinal	Gasteroid fungi	
*Geastrum triplex* Jungh.		√			√	√	LS	Medicinal	Gasteroid fungi	
*Gerronema albidum* (Fr.) Singer	√					√	SS	Edible	Agarics	
*Gliophorus psittacinu* (Schaeff.) Herink						√	SS	Edible, Poisonous	Agarics	
*Gloeophyllum sepiarium* (Wulfen) P. Karst.		√					WS	Medicinal	PHT fungi	
*Gloeophyllum subferrugineum* (Berk.) Bondartsev and Singer	√			√			WS	Others	PHT fungi	
*Gloeophyllum trabeum* (Pers.) Murrill					√		WS	Medicinal	PHT fungi	
*Gloeostereum incarnatum* S. Ito and S. Imai		√			√	√	WS	Edible, Medicinal	PHT fungi	
*Gomphidius maculatus* (Scop.) Fr.		√					EM	Edible	Boletes	
*Gomphus clavatus* (Pers.) Gray					√		EM	Edible, Medicinal	Cantharelloid fungi	
*Guepinia helvelloides* (DC.) Fr.	√			√			SS	Edible	Jelly fungi	
*Gymnopilus aeruginosus* (Peck) Singer		√	√				WS	Others	Agarics	
*Gymnopilus junonius* (Fr.) P.D. Orton		√					WS	Others	Agarics	
*Gymnopilus liquiritiae* (Pers.) P. Karst.		√			√		WS	Medicinal, Poisonous	Agarics	
*Gymnopilus penetrans* (Fr.) Murrill	√						WS	Poisonous	Agarics	ON683447
*Gymnopus alkalivirens* (Singer) Halling					√		LS	Others	Agarics	
*Gymnopus androsaceus* (L.) J.L. Mata and R.H. Petersen			√		√	√	LS	Medicinal	Agarics	
*Gymnopus dryophilus* (Bull.) Murril	√	√	√	√	√	√	LS	Edible, Poisonous	Agarics	ON683448
*Gymnopus erythropus* (Pers.) Antonín, Halling and Noordel.				√			LS	Edible	Agarics	ON683449
*Gymnopus fusipes* (Bull.) Gray					√	√	LS	Edible	Agarics	
*Gymnopus ocior* (Pers.) Antonín and Noordel.						√	LS	Edible	Agarics	
*Gymnopus polyphyllus* (Peck) Halling	√						LS	Others	Agarics	
*Harrya chromipes* (Frost) Halling, Nuhn, Osmundson and Manfr. Binder					√		EM	Others	Boletes	
*Hebeloma hiemale* Bres.			√				SS	Others	Agarics	
*Hebeloma radicosum* (Bull.) Ricken			√				SS	Edible, Poisonous	Agarics	
*Helvella atra* J. König						√	SS	Edible	Larger Ascomycetes	
*Helvella crispa* (Scop.) Fr.	√	√	√	√	√	√	SS	Edible, Poisonous	Larger Ascomycetes	
*Helvella elastica* Bull.	√		√	√	√	√	SS	Edible, Poisonous	Larger Ascomycetes	
*Helvella ephippium* Lév.					√	√	SS	Edible	Larger Ascomycetes	
*Helvella lacunosa* Afzel.			√			√	SS	Edible, Medicinal	Larger Ascomycetes	
*Hemistropharia albocrenulata* (Peck) Jacobsson and E. Larss.						√	WS	Edible, Poisonous	Agarics	
*Hericium coralloides* (Scop.) Pers.		√					WS	Edible, Medicinal	PHT fungi	
*Hericium erinaceus* (Bull.) Pers.	√	√	√				WS	Edible, Medicinal	PHT fungi	ON683494
*Heterobasidion insulare* (Murrill) Ryvarden		√	√				WS	Others	PHT fungi	
*Hohenbuehelia reniformis* (G. Mey.) Singer		√				√	WS	Edible	Agarics	
*Hohenbuehelia serotina* (Pers.) Singer				√			WS	Others	Agarics	
*Hortiboletus rubellus* (Krombh.) Simonini, Vizzini and Gelardi	√					√	EM	Edible, Medicinal	Boletes	
*Humaria hemisphaerica* (F.H. Wigg.) Fuckel	√		√		√	√	SS	Others	Larger Ascomycetes	
*Hydnum repandum* L.			√	√	√	√	EM	Edible, Medicinal	PHT fungi	
*Hygrocybe ceracea* (Sowerby) P. Kumm.	√			√			SS	Edible	Agarics	
*Hygrocybe chlorophana* (Fr.) Wünsche			√				SS	Edible	Agarics	
*Hygrocybe coccinea* (Schaeff.) P. Kumm.			√				SS	Edible	Agarics	
*Hygrocybe conica* (Schaeff.) P. Kumm.		√	√	√	√		SS	Medicinal, Poisonous	Agarics	
*Hygrocybe flavescens* (Kauffman) Singer			√				SS	Poisonous	Agarics	
*Hygrocybe marchii* (Bres.) Singer	√			√			SS	Others	Agarics	
*Hygrocybe miniata* (Fr.) P. Kumm.	√		√			√	SS	Edible	Agarics	ON683450
*Hygrophorus chrysodon* (Batsch) Fr.	√				√	√	SS	Edible	Agarics	
*Hygrophorus eburneus* (Bull.) Fr.			√	√			SS	Edible	Agarics	
*Hygrophorus laurae* Morgan	√					√	SS	Others	Agarics	
*Hygrophorus lucorum* Kalchbr.	√	√			√		SS	Edible, Medicinal	Agarics	
*Hygrophorus occidentalis* A.H. Sm. and Hesler	√		√				SS	Others	Agarics	
*Hygrophorus olivaceo-albus* (Fr.) Fr.			√				SS	Edible	Agarics	
*Hygrophorus piceae* Kühner	√			√			SS	Others	Agarics	
*Hygrophorus pseudochrysaspis* Hesler and A.H. Sm.	√		√		√		SS	Others	Agarics	
*Hygrophorus russula* (Schaeff. ex Fr.) Kauffman	√		√				SS	Edible	Agarics	ON683451
*Hymenochaete adusta* (Lév.) Har. and Pat.						√	WS	Others	PHT fungi	
*Hymenochaete corrugata* (Fr.) Lév.	√	√				√	WS	Others	PHT fungi	
*Hymenochaete xerantica* (Berk.) S.H. He and Y.C. Dai						√	WS	Others	PHT fungi	
*Hymenopellis radicata* (Relhan) R.H. Petersen					√	√	SS	Others	Agarics	
*Hymenoscyphus fructigenus* (Bull.) Gray						√	WS	Others	Larger Ascomycetes	
*Hypholoma capnoides* (Fr.) P. Kumm.		√					WS	Medicinal, Poisonous	Agarics	
*Hypholoma fasciculare* (Huds.) P. Kumm.		√	√	√	√	√	WS	Medicinal, Poisonous	Agarics	
*Hypholoma lateritium* (Schaeff.) P. Kumm.		√	√				WS	Poisonous	Agarics	
*Hypsizygus marmoreus* (Peck) H.E. Bigelow		√					WS	Others	Agarics	ON683452
*Hypsizygus ulmarius* (Bull.) Redhead		√		√			WS	Edible, Medicinal	Agarics	
*Infundibulicybe geotropa* (Bull.) Harmaja	√						SS	Edible, Medicinal, Poisonous	Agarics	
*Infundibulicybe gibba* (Pers.) P. Kumm.	√	√		√	√		LS	Edible	Agarics	ON683453
*Inocybe asterospora* Quél.					√	√	EM	Poisonous	Agarics	
*Inocybe calamistrata* (Fr.) Gillet					√		EM	Poisonous	Agarics	
*Inocybe changbaiensis* T. Bau and Y.G. Fan	√			√			EM	Others	Agarics	
*Inocybe cookei* Bres.				√			EM	Poisonous	Agarics	
*Inocybe earleana* Kauffma	√			√			EM	Others	Agarics	
*Inocybe euviolacea* E. Ludw.						√	EM	Others	Agarics	
*Inocybe fulvella* Bres.	√						EM	Others	Agarics	
*Inocybe geophylla* P. Kumm.	√		√		√	√	EM	Poisonous	Agarics	
*Inocybe napipes* J.E. Lange	√				√		EM	Poisonous	Agarics	
*Inocybe praetervisa* Quél.					√		EM	Poisonous	Agarics	
*Inocybe subalbidodisca* Stangl and J. Veselský	√				√		EM	Others	Agarics	
*Inocybe umbrinella* Bres.	√			√	√		EM	Others	Agarics	
*Inonotus hispidus* (Bull.) P. Karst.	√						WS	Medicinal	PHT fungi	ON683454
*Inonotus obliquus* (Fr.) Pilát				√			WS	Medicinal	PHT fungi	
*Inosperma calamistratum* (Fr.) Matheny and Esteve-Rav.						√	LS	Others	Agarics	
*Irpex lacteus* (Fr.) Fr.		√			√		WS	Others	PHT fungi	
*Isaria japonica* Yasuda						√	EI	Others	Larger Ascomycetes	
*Junghuhnia nitida* (Pers.) Ryvarden						√	WS	Edible	PHT fungi	
*Kuehneromyces mutabilis* (Schaeff.) Singer and A.H. Sm.	√	√	√		√		WS	Edible	Agarics	ON683455
Laccaria alba Zhu L. Yang and Lan Wang	√			√			EM	Edible	Agarics	ON683456
*Laccaria amethystea* (Bull.) Murrill			√	√		√	EM	Others	Agarics	ON683457
*Laccaria amethystina* Cooke	√						EM	Others	Agarics	
*Laccaria laccata* (Scop.) Cooke	√		√	√	√		EM	Edible, Medicinal	Agarics	ON683458
*Laccaria proxima* (Boud.) Pat.	√		√				EM	Others	Agarics	
*Laccaria purpureobadia* D.A. Reid				√			EM	Others	Agarics	
*Laccaria tortilis* (Bolton) Cooke	√						EM	Edible, Medicinal	Agarics	ON683459
*Lacrymaria lacrymabunda* (Bull.) Pat.					√		SS	Poisonous	Agarics	
*Lactarius acris* (Bolton) Gray				√			EM	Others	Agarics	
*Lactarius aurantiacus* (Pers.) Gray	√		√				EM	Edible, Medicinal	Agarics	
*Lactarius camphoratus* (Bull.) Fr.			√		√		EM	Edible	Agarics	
*Lactarius circellatus* Fr.			√				EM	Edible	Agarics	
*Lactarius deliciosus* (L.) Gray	√				√	√	EM	Edible, Medicinal	Agarics	
*Lactarius fuliginosus* (Fr.) Fr.			√				EM	Edible	Agarics	
*Lactarius hatsudake* Nobuj. Tanaka	√		√			√	EM	Edible, Medicinal	Agarics	
*Lactarius lignyotus* Fr.					√		EM	Medicinal, Poisonous	Agarics	
*Lactarius mitissimus* (Fr.) Fr.			√				EM	Others	Agarics	
*Lactarius piperatus* (L.) Pers.	√		√	√	√	√	EM	Edible, Medicinal, Poisonous	Agarics	
*Lactarius subdulcis* (Pers.) Gray	√						EM	Edible	Agarics	
*Lactarius theiogalus* (Bull.) Gray	√						EM	Others	Agarics	
*Lactarius torminosus* (Schaeff.) Pers.			√			√	EM	Poisonous	Agarics	
*Lactarius trivialis* (Fr.) Fr.					√		EM	Others	Agarics	
*Lactarius vellereus* (Fr.) Fr.	√		√				EM	Edible, Medicinal, Poisonous	Agarics	
*Lactarius vietus* (Fr.) Fr.	√		√	√			EM	Edible	Agarics	
*Lactarius volemus* (Fr.) Fr.	√		√				EM	Edible, Medicinal	Agarics	ON683460
*Lactarius zonarius* (Bull.) Fr.	√				√		EM	Medicinal, Poisonous	Agarics	
*Lactifluus subpiperatus* (Hongo) Verbeken	√				√		EM	Others	Agarics	
*Laetiporus cremeiporus* Y. Ota and T. Hatt.		√					WS	Edible, Medicinal	PHT fungi	
*Laetiporus montanus* Černý ex Tomšovský and Jankovský		√					WS	Edible	PHT fungi	
*Laetiporus sulphureus* (Bull.) Murrill			√	√	√		WS	Edible, Medicinal	PHT fungi	ON683461
*Leccinum chromapes* (Frost) Singer			√			√	EM	Others	Boletes	
*Leccinum scabrum* (Bull.) Gray	√		√		√	√	EM	Edible, Poisonous	Boletes	
*Lentinellus flabelliformis* (Bolton) S. Ito	√	√					WS	Others	Agarics	
*Lentinellus ursinus* (Fr.) Kühner		√			√	√	WS	Edible	Agarics	
*Lentinula edodes* (Berk.) Pegler		√					WS	Edible, Medicinal	Agarics	
*Lentinus arcularius* (Batsch) Zmitr.	√	√	√		√	√	WS	Medicinal	PHT fungi	
*Lentinus brumalis* (Pers.) Zmitr.	√						WS	Edible	PHT fungi	
*Lentinus elmeri* Bres.			√				WS	Others	PHT fungi	
*Lentinus substrictus* (Bolton) Zmitr. and Kovalenko		√		√			WS	Others	PHT fungi	
*Lentinus tigrinus* (Bull.) Fr.		√		√			WS	Edible, Medicinal	PHT fungi	
*Lenzites albida* (Fr.) Fr.			√				WS	Edible	PHT fungi	
*Lenzites betulinus* (L.) Fr.		√			√	√	WS	Medicinal	PHT fungi	
*Lenzites repanda* Fr.			√				WS	Poisonous	PHT fungi	
*Leotia lubrica* (Scop.) Pers.			√			√	SS	Others	Larger Ascomycetes	
*Lepiota brunneoincarnata* Chodat and C. Martín						√	LS	Poisonous	Agarics	
*Lepiota castanea* Quél.	√			√	√	√	LS	Poisonous	Agarics	
*Lepiota clypeolaria* (Bull.) P. Kumm.	√				√	√	LS	Poisonous	Agarics	ON683462
*Lepiota cristata* (Bolton) P. Kumm.	√		√	√	√	√	LS	Poisonous	Agarics	
*Lepiota erminea* (Fr.) P. Kumm.	√					√	LS	Edible	Agarics	
Lepiota felina (Pers.) P. Karst.	√		√		√		LS	Others	Agarics	
*Lepiota fusciceps* Hongo						√	LS	Others	Agarics	
*Lepiota magnispora* Murrill				√			LS	Others	Agarics	
*Lepista glaucocana* (Bres.) Singer	√					√	LS	Edible	Agarics	
*Lepista irina* (Fr.) H.E. Bigelow	√	√	√				LS	Medicinal, Poisonous	Agarics	
*Lepista nuda* (Bull.) Cooke	√	√	√	√	√	√	LS	Edible, Medicinal	Agarics	ON683463
*Lepista personata* (Fr.) Cooke	√				√		LS	Edible, Medicinal	Agarics	
*Lepista sordida* (Schumach.) Singer		√			√	√	LS	Edible, Medicinal	Agarics	
*Leucoagaricus leucothites* (Vittad.) Wasser	√					√	SS	Edible, Medicinal, Poisonous	Agarics	
*Leucoagaricus rubrotinctus* (Peck) Singer	√		√		√		SS	Others	Agarics	ON683464
*Leucocoprinus brebissonii* (Godey) Locq.	√			√			SS	Others	Agarics	
*Leucocybe candicans* (Pers.) Vizzini, P. Alvarado, G. Moreno, and Consiglio						√	SS	Medicinal, Poisonous	Agarics	
*Leucocybe connata* (Schumach.) Vizzini, P. Alvarado, G. Moreno, and Consiglio			√		√		SS	Edible, Poisonous	Agarics	
*Leucocybe houghtonii* (W. Phillips) Halama and Pencak.				√			SS	Others	Agarics	
*Lopharia cinerascens* (Schwein.) G. Cunn.						√	WS	Others	PHT fungi	
*Lycoperdon fuscum* Huds.						√	SS	Edible, Medicinal	Gasteroid fungi	
*Lycoperdon mammaeforme* Pers.			√		√	√	SS	Others	Gasteroid fungi	
*Lycoperdon pedicellatum* Batsch					√		SS	Edible, Medicinal	Gasteroid fungi	
*Lycoperdon perlatum* Pers.	√	√			√	√	SS	Edible, Medicinal	Gasteroid fungi	ON683465
*Lycoperdon pusillum* Hedw.					√		SS	Others	Gasteroid fungi	
*Lycoperdon pyriforme* Schaeff.		√					SS	Edible, Medicinal	Gasteroid fungi	
*Lycoperdon umbrinum* Pers.	√		√		√		SS	Edible, Medicinal	Gasteroid fungi	
*Lyophyllum decastes* (Fr.) Singer		√			√		EM	Edible, Medicinal	Agarics	
*Lysurus mokusin* (L.) Fr.					√	√	SS	Medicinal	Gasteroid fungi	
*Macrocystidia cucumis* (Pers.) Joss.			√			√	SS	Others	Agarics	
*Macrolepiota procera* (Scop.) Singer	√	√	√	√	√	√	SS	Edible, Medicinal, Poisonous	Agarics	
*Mallocybe terrigena* (Fr.) Matheny, Vizzini, and Esteve-Rav.				√			SS	Edible	Agarics	
*Marasmiellus candidus* (Fr.) Singer					√		WS	Others	Agarics	
*Marasmiellus eburneus* (Theiss.) Singer	√		√				WS	Others	Agarics	
*Marasmiellus ramealis* (Bull.) Singer	√			√	√	√	WS	Medicinal	Agarics	
*Marasmius aurantiacus* I. Hino					√		LS	Edible, Medicinal	Agarics	
*Marasmius beniensis* Singer			√				LS	Others	Agarics	
*Marasmius chordalis* Fr.	√				√		LS	Others	Agarics	
*Marasmius cohaerens* (Pers.) Cooke and Quél.	√		√		√	√	LS	Others	Agarics	
*Marasmius epiphyllus* (Pers.) Fr.			√				LS	Others	Agarics	
*Marasmius floriceps* Berk. and M.A. Curtis	√						LS	Others	Agarics	
*Marasmius maximus* Hongo	√		√		√		LS	Edible	Agarics	ON683427
*Marasmius occultatiformis* Antonín, Ryoo, and H.D. Shin	√				√		LS	Others	Agarics	ON683419
*Marasmius oreades* (Bolton) Fr.	√				√	√	LS	Edible, Medicinal	Agarics	
*Marasmius pallidocephalus* Gilliam	√			√			LS	Others	Agarics	
*Marasmius polylepidis* Dennis	√			√			LS	Others	Agarics	
*Marasmius pseudoniveus* Singer			√				LS	Others	Agarics	
*Marasmius pulcherripes* Peck						√	LS	Others	Agarics	
*Marasmius riparius* Singer	√		√		√		LS	Others	Agarics	
*Marasmius rotuloides* Dennis	√			√			LS	Others	Agarics	
*Marasmius sessiliaffinis* Singer			√				LS	Others	Agarics	
*Marasmius siccus* (Schwein.) Fr.	√		√			√	LS	Others	Agarics	
*Megacollybia platyphylla* (Pers.) Kotl. and Pouzar			√				WS	Others	Agarics	
*Melanoleuca brevipes* (Bull.) Pat.						√	SS	Edible	Agarics	
*Melanoleuca grammopodia* (Bull.) Murrill	√		√				SS	Edible	Agarics	
*Melanoleuca melaleuca* (Pers.) Murrill	√				√	√	SS	Edible	Agarics	ON683466
*Melanoleuca strictipes* (P. Karst.) Jul. Schäff.	√						SS	Edible	Agarics	
*Melanoleuca stridula* (Fr.) Singer					√	√	SS	Others	Agarics	
*Melanoleuca verrucipes* (Fr.) Singer	√			√		√	SS	Edible	Agarics	
*Melastiza chateri* (W.G. Sm.) Boud.						√	SS	Others	Larger Ascomycetes	
*Microporus affinis* (Blume and T. Nees) Kuntze	√						WS	Others	PHT fungi	
*Microstoma floccosum* (Sacc.) Raitv.		√					WS	Others	Larger Ascomycetes	
*Morchella conica* Pers.						√	SS	Edible	Larger Ascomycetes	
*Morchella crassipes* (Vent.) Pers.					√	√	SS	Edible, Medicinal	Larger Ascomycetes	
*Morchella esculenta* (L.) Pers.	√		√		√	√	SS	Edible, Medicinal	Larger Ascomycetes	
*Morchella vulgaris* (Pers.) Gray			√				SS	Edible, Medicinal	Larger Ascomycetes	
*Mucidula brunneomarginata* (Lj.N. Vassiljeva) R.H. Petersen		√	√	√			WS	Edible	Agarics	
*Mucidula mucida* (Schrad.) Pat.	√	√	√	√			WS	Others	Agarics	ON683467
*Mutinus caninus* (Huds.) Fr.			√			√	SS	Poisonous	Gasteroid fungi	
*Mycena alphitophora* (Berk.) Sacc.						√	LS	Others	Agarics	
*Mycena collybiiformis* (Murrill) Murrill	√						LS	Others	Agarics	
*Mycena debilis* (Fr.) Quél.	√		√				LS	Others	Agarics	
*Mycena epipterygia* (Scop.) Gray	√						LS	Others	Agarics	
*Mycena filopes* (Bull.) P. Kumm.					√		LS	Others	Agarics	
*Mycena flavescens* Velen.	√			√			LS	Poisonous	Agarics	
*Mycena fuliginella* A.H. Sm.	√		√				LS	Others	Agarics	
*Mycena galericulata* (Scop.) Gray	√		√		√		LS	Edible, Medicinal	Agarics	ON683420
*Mycena haematopus* (Pers.) P. Kumm.	√	√	√	√	√		LS	Medicinal, Poisonous	Agarics	ON683468
*Mycena inclinata* (Fr.) Quél.	√						LS	Others	Agarics	
*Mycena nucleata* X. He and X.D. Fang	√			√			LS	Others	Agarics	
*Mycena osmundicola* J.E. Lange			√				LS	Others	Agarics	
*Mycena pseudoandrosacea* (Bull.) Z.S. Bi	√		√				LS	Others	Agarics	
*Mycena pura* (Pers.) P. Kumm.	√	√	√	√	√	√	LS	Medicinal, Poisonous	Agarics	ON683469
*Mycena rosea* Gramberg	√			√			LS	Others	Agarics	
*Mycena sanguinolenta* (Alb. and Schwein.) P. Kumm.	√						LS	Others	Agarics	
*Mycena subcana* A.H. Sm.	√						LS	Others	Agarics	
*Mycena subgracilis* Métrod	√			√			LS	Others	Agarics	
*Mycoleptodonoides pergamenea* (Yasuda) Aoshima and H. Furuk.		√		√			WS	Others	PHT fungi	
*Myxarium nucleatum* Wallr.	√		√	√			WS	Others	Larger Ascomycetes	
*Naematelia aurantialba* (Bandoni and M. Zang) Millanes and Wedin	√		√				WS	Edible, Medicinal	Jelly fungi	
*Neofavolus alveolaris* (DC.) Sotome and T. Hatt.			√				WS	Others	PHT fungi	ON683470
*Neolentinus adhaerens* (Alb. and Schwein.) Redhead and Ginns		√					WS	Edible, Medicinal	PHT fungi	
*Neolentinus cyathiformis* (Schaeff.) Della Magg. and Trassin.		√					WS	Others	PHT fungi	
*Neolentinus lepideus* (Fr.) Redhead and Ginns	√	√	√	√	√	√	WS	Edible, Medicinal, Poisonous	PHT fungi	ON683471
*Nidula niveotomentosa* (Henn.) Lloyd						√	WS	Medicinal	Agarics	
*Omphalina lilaceorosea* Svrček and Kubička		√					LS	Others	Agarics	
*Omphalotus guepiniformis* (Berk.) Neda	√	√	√				WS	Poisonous	Agarics	
*Onnia tomentosa* (Fr.) P. Karst.					√		WS	Others	PHT fungi	
*Ophiocordyceps nutans* (Pat.) G.H. Sung, J.M. Sung, Hywel-Jones, and Spatafora				√			EI	Medicinal	Larger Ascomycetes	
*Ophiocordyceps sphecocephala* (Klotzsch ex Berk.) G.H. Sung, J.M. Sung, Hywel-Jones, and Spatafora		√				√	EI	Medicinal	Larger Ascomycetes	
*Ossicaulis lignatilis* (Pers.) Redhead and Ginns		√				√	WS	Edible	Agarics	
*Osteina undosa* (Peck) Zmitr.						√	WS	Others	PHT fungi	
*Otidea cochleata* (L.) Fuckel	√		√	√	√	√	SS	Poisonous	Larger Ascomycetes	
*Otidea leporina* (Batsch) Fuckel				√			SS	Others	Larger Ascomycetes	
*Oudemansiella radicata* (Relhan) Singer		√					SS	Others	Agarics	
*Panaeolus campanulatus* (L.) Quél.			√				DS	Others	Agarics	
*Panaeolus fimicola* (Pers.) Gillet	√		√				DS	Poisonous	Agarics	
*Panaeolus papilionaceus* (Bull.) Quél.	√				√	√	DS	Poisonous	Agarics	
*Panellus stipticus* (Bull.) P. Karst.	√	√			√		WS	Medicinal, Poisonous	Agarics	ON683472
*Panus conchatus* (Bull.) Fr.		√					WS	Edible, Medicinal	Agarics	ON673473
*Panus rudis* Fr.					√	√	WS	Others	Agarics	
*Paralepista splendens* (Pers.) Vizzini	√		√		√		SS	Others	Agarics	
*Paralepistopsis acromelalga* (Ichimura) Vizzini						√	SS	Poisonous	Agarics	
*Parasola plicatilis* (Curtis) Redhead, Vilgalys, and Hopple	√			√			SS	Medicinal	Agarics	
*Parasola leiocephala* (P.D. Orton) Redhead, Vilgalys and Hopple	√	√					SS	Others	Agarics	
*Paxillus involutus* (Batsch) Fr.		√	√		√	√	EM	Medicinal, Poisonous	Boletes	
*Perenniporia inflexibilis* (Berk.) Ryvarden	√			√			WS	Others	PHT fungi	
*Perenniporia medulla-panis* (Jacq.) Donk			√			√	WS	Others	PHT fungi	
*Perenniporia subacida* (Peck) Donk			√			√	WS	Medicinal	PHT fungi	
*Peziza ampliata* Pers.			√				SS	Others	Larger Ascomycetes	
*Peziza badia* Pers.		√		√	√	√	SS	Poisonous	Larger Ascomycetes	
*Peziza praetervisa* Bres.	√		√			√	SS	Others	Larger Ascomycetes	
*Peziza sylvestris* (Boud.) Sacc. and Traverso					√		SS	Others	Larger Ascomycetes	
*Peziza vesiculosa* Bolton		√	√		√	√	SS	Edible, Poisonous	Larger Ascomycetes	
*Phaeoclavulina abietina* (Pers.) Giachini			√				LS	Others	Coral fungi	
*Phaeoclavulina flaccida* (Fr.) Giachini			√				LS	Poisonous	Coral fungi	
*Phaeolepiota aurea* (Matt.) Maire				√	√		SS	Edible, Medicinal, Poisonous	Agarics	
*Phaeolus schweinitzii* (Fr.) Pat.					√		WS	Medicinal	PHT fungi	
*Phaeotremella foliacea* (Pers.) Wedin, J.C. Zamora and Millanes			√		√		EM	Edible, Medicinal	Jelly fungi	
*Phallus flavocostatus* Kreisel	√		√				SS	Others	Gasteroid fungi	ON683474
*Phallus hadriani* Vent.						√	SS	Others	Gasteroid fungi	
*Phallus impudicus* L.	√				√	√	SS	Edible, Medicinal	Gasteroid fungi	
*Phallus indusiatus* Vent.					√		SS	Others	Gasteroid fungi	
*Phallus rubicundus* (Bosc) Fr.	√						SS	Medicinal, Poisonous	Gasteroid fungi	
*Phallus rugulosus* (E. Fisch.) Lloyd					√	√	SS	Others	Gasteroid fungi	
*Phellinus gilvus* (Schwein.) Pat.					√	√	WS	Others	PHT fungi	
*Phellinus igniarius* (L.) Quél.		√					WS	Medicinal	PHT fungi	
*Phellodon fuligineoalbus* (J.C. Schmidt) R.E. Baird			√				WS	Others	PHT fungi	
*Phellodon tomentosus* (L.) Banker	√						WS	Edible	PHT fungi	
*Phillipsia domingensis* (Berk.) Berk. ex Denison		√	√				WS	Others	Larger Ascomycetes	
*Phlebia tremellosa* (Schrad.) Nakasone and Burds.					√		WS	Medicinal	PHT fungi	
*Phloeomana minutula* (Sacc.) Redhead	√						WS	Others	Agarics	
*Phloeomana speirea* (Fr.) Redhead			√		√	√	WS	Others	Agarics	
*Pholiota adiposa* (Batsch) P. Kumm.	√	√		√		√	WS	Edible, Medicinal	Agarics	
*Pholiota aurivella* (Batsch) P. Kumm.		√	√	√	√	√	WS	Edible, Medicinal	Agarics	
*Pholiota flammans* (Batsch) P. Kumm.	√	√	√		√		WS	Edible, Medicinal, Poisonous	Agarics	
*Pholiota hiemalis* A.H. Sm. and Hesler		√		√			WS	Others	Agarics	
*Pholiota lenta* (Pers.) Singer			√				WS	Edible, Medicinal	Agarics	
*Pholiota lubrica* (Pers.) Singer	√						WS	Edible, Medicinal, Poisonous	Agarics	ON683475
*Pholiota mutabilis* (Schaeff.) P. Kumm.					√		WS	Others	Agarics	
*Pholiota spumosa* (Fr.) Singer	√			√	√		WS	Edible, Medicinal	Agarics	ON683476
*Pholiota squarrosa* (Vahl) P. Kumm.	√	√	√		√		WS	Edible, Medicinal, Poisonous	Agarics	
*Pholiota squarrosoides* (Peck) Sacc.	√	√	√		√		WS	Edible, Poisonous	Agarics	ON683477
*Pholiota subflavida* (Murrill) A.H. Sm. and Hesler	√				√		WS	Others	Agarics	
*Pholiota tuberculosa* (Schaeff.) P. Kumm.			√				WS	Others	Agarics	
*Pholiota veris* A.H. Sm. and Hesler						√	WS	Others	Agarics	
*Pholiota vinaceobrunnea* A.H. Sm. and Hesler		√					WS	Others	Agarics	
*Pholiota terrestris* Overh.	√	√	√		√		WS	Edible, Medicinal, Poisonous	Agarics	
*Phyllotopsis nidulans* (Pers.) Singer			√			√	WS	Edible	Agarics	
*Physalacria lateriparies* X. He and F.Z. Xue					√		WS	Others	Agarics	
*Picipes badius* (Pers.) Zmitr. and Kovalenko			√			√	WS	Others	PHT fungi	ON683478
*Picipes melanopus* (Pers.) Zmitr. and Kovalenko			√				WS	Others	PHT fungi	
*Piptoporus betulinus* (Bull.) P. Karst.		√		√			WS	Medicinal	PHT fungi	
*Plectania melastoma* (Sowerby) Fuckel	√			√			WS	Others	Larger Ascomycetes	
*Pleuroflammula flammea* (Murrill) Singer		√					SS	Others	Agarics	
*Pleuroflammula multifolia* (Peck) E. Horak	√						SS	Others	Agarics	
*Pleurotus citrinopileatus* Singer	√	√	√				WS	Edible, Medicinal	Agarics	ON683479
*Pleurotus cornucopiae* (Paulet) Quél.	√	√	√		√		WS	Edible, Medicinal	Agarics	
*Pleurotus corticatus* (Fr.) P. Kumm.			√				WS	Others	Agarics	
*Pleurotus dryinus* (Pers.) P. Kumm.	√			√			WS	Edible, Medicinal	Agarics	
*Pleurotus limpidus* (Fr.) P. Karst.	√				√		WS	Edible	Agarics	
*Pleurotus ostreatus* (Jacq.) P. Kumm.	√	√	√	√	√	√	WS	Edible, Medicinal	Agarics	ON683480
*Pleurotus pulmonarius* (Fr.) Quél.	√	√	√		√	√	WS	Edible, Medicinal	Agarics	ON683481
*Pleurotus spodoleucus* (Fr.) Quél.	√			√			WS	Edible, Medicinal	Agarics	
*Pluteus atricapillus* (Batsch) Fayod			√				WS	Others	Agarics	
*Pluteus atromarginatus* (Konrad) Kühner						√	WS	Edible	Agarics	
*Pluteus aurantiorugosus* (Trog) Sacc.		√	√		√		WS	Edible	Agarics	ON683482
*Pluteus cervinus* (Schaeff.) P. Kumm.	√	√		√	√	√	WS	Edible	Agarics	
*Pluteus depauperatus* Romagn.			√				WS	Others	Agarics	
*Pluteus leoninus* (Schaeff.) P. Kumm.		√	√		√		WS	Edible	Agarics	
*Pluteus nanus* (Pers.) P. Kumm.			√		√		WS	Others	Agarics	
*Pluteus petasatus* (Fr.) Gillet					√	√	WS	Edible	Agarics	
*Pluteus plautus* (Weinm.) Gillet	√	√					WS	Others	Agarics	
*Pluteus salicinus* (Pers.) P. Kumm.		√	√				WS	Edible	Agarics	
*Pluteus umbrosus* (Pers.) P. Kumm.	√						WS	Edible	Agarics	
*Podosordaria pedunculata* (Dicks.) Dennis		√		√			WS	Others	Larger Ascomycetes	
*Polyporus alveolaris* (DC.) Bondartsev and Singer					√	√	WS	Others	PHT fungi	
*Polyporus badius* (Pers.) Schwein.					√		WS	Others	PHT fungi	
*Polyporus brumalis* (Pers.) Fr.		√					WS	Others	PHT fungi	
*Polyporus conifericola* H.J. Xue and L.W. Zhou		√					WS	Others	PHT fungi	ON683483
*Polyporus squamosus* (Huds.) Fr.		√					WS	Medicinal	PHT fungi	
*Polyporus tuberaster* (Jacq. ex Pers.) Fr.		√		√			WS	Others	PHT fungi	
*Polyporus varius* (Pers.) Fr.		√					WS	Medicinal	PHT fungi	
*Polystictus unicolor* Rick	√				√		WS	Others	PHT fungi	
*Polystictus versicolor* (L.) Fr.					√		WS	Others	PHT fungi	
*Psathyrella candolleana* (Fr.) Maire	√	√			√	√	WS	Medicinal, Poisonous	Agarics	ON683484
*Psathyrella hydrophila* (Bull.) Maire					√		WS	Others	Agarics	
*Psathyrella multissima* (S. Imai) Hongo		√	√		√	√	WS	Others	Agarics	
*Psathyrella subnuda* (P. Karst.) A.H. Sm.		√	√				WS	Others	Agarics	
*Pseudoclitocybe cyathiformis* (Bull.) Singer	√				√	√	SS	Edible, Medicinal	Agarics	ON683485
*Pseudofavolus tenuis* (Fr.) G. Cunn.	√			√			WS	Medicinal, Poisonous	PHT fungi	
*Pseudohydnum gelatinosum* (Scop.) P. Karst.				√			WS	Edible, Medicinal	Jelly fungi	
*Pseudosperma avellaneum* (Kobayasi) Matheny and Esteve-Rav.	√						SS	Others	Agarics	
*Pseudosperma rimosum* (Bull.) Matheny and Esteve-Rav.	√		√	√	√	√	SS	Others	Agarics	
*Pseudosperma umbrinellum* (Bres.) Matheny and Esteve-Rav.						√	SS	Others	Agarics	
*Pterula multifida* (Chevall.) Fr.		√	√		√	√	SS	Others	Coral fungi	ON683486
*Pycnoporus cinnabarinus* (Jacq.) P. Karst.	√	√				√	WS	Medicinal	PHT fungi	
*Pycnoporus sanguineus* (L.) Murrill					√		WS	Medicinal	PHT fungi	
*Radulodon copelandii* (Pat.) N. Maek.			√			√	WS	Others	PHT fungi	
*Radulomyces copelandii* (Pat.) Hjortstam and Spooner		√	√				WS	Others	PHT fungi	
*Ramaria apiculata* (Fr.) Donk	√	√	√		√	√	EM	Edible, Medicinal	Coral fungi	
*Ramaria botrytis* (Pers.) Bourdot					√		EM	Edible, Medicinal	Coral fungi	
*Ramaria bourdotiana* Maire			√				EM	Edible	Coral fungi	
*Ramaria flava* (Schaeff.) Quél.	√		√		√	√	EM	Edible, Medicinal, Poisonous	Coral fungi	
*Ramaria formosa* (Pers.) Quél.			√				EM	Edible, Medicinal, Poisonous	Coral fungi	
*Ramaria lutea* Schild						√	EM	Edible	Coral fungi	
*Ramaria madagascariensis* (Henn.) Corner			√				EM	Others	Coral fungi	
*Ramaria stricta* (Pers.) Quél.	√		√		√	√	EM	Edible	Coral fungi	
*Ramaria subbotrytis* (Coker) Corner			√				EM	Edible	Coral fungi	
*Ramariopsis kunzei* (Fr.) Corner						√	EM	Edible	Coral fungi	
*Resupinatus applicatus* (Batsch) Gray		√				√	WS	Others	Agarics	
*Rhizocybe vermicularis* (Fr.) Vizzini, P. Alvarado, G. Moreno, and Consiglio			√				SS	Others	Agarics	
*Rhizomarasmius undatus* (Berk.) R.H. Petersen						√	SS	Others	Agarics	
*Rhodocollybia butyracea* (Bull.) Lennox		√					SS	Edible	Agarics	
*Rhodocollybia prolixa* (Fr.) Antonín and Noordel.						√	SS	Others	Agarics	
*Rickenella fibula* (Bull.) Raithelh.	√		√				EM	Others	Cantharelloid fungi	
*Ripartites tricholoma* (Alb. and Schwein.) P. Karst.	√						LS	Others	Agarics	ON683428
*Russula adusta* (Pers.) Fr.	√						EM	Edible, Medicinal	Agarics	
*Russula aeruginea* Lindblad ex Fr.	√		√		√	√	EM	Edible	Agarics	
*Russula albida* A. Blytt	√		√				EM	Edible	Agarics	
*Russula alutacea* (Fr.) Fr.					√		EM	Edible, Medicinal, Poisonous	Agarics	
*Russula amoena* Quél.	√		√	√		√	EM	Others	Agarics	
*Russula aurata* Fr.			√		√		EM	Others	Agarics	
*Russula aurea* Pers.	√						EM	Others	Agarics	
*Russula chloroides* (Krombh.) Bres.	√		√				EM	Edible	Agarics	
*Russula crustosa* Peck	√		√				EM	Edible, Medicinal	Agarics	ON683429
*Russula cyanoxantha* (Schaeff.) Fr.	√		√			√	EM	Edible, Medicinal	Agarics	ON683430
*Russula delica* Fr.	√				√	√	EM	Edible, Medicinal	Agarics	
*Russula densifolia* Secr. ex Gillet			√			√	EM	Edible, Medicinal	Agarics	ON683487
*Russula emetica* (Schaeff.) Pers.	√		√		√		EM	Edible, Medicinal, Poisonous	Agarics	
*Russula exalbicans* (Pers.) Melzer and Zvára				√			EM	Edible	Agarics	
*Russula faginea* Romagn.			√				EM	Edible	Agarics	
*Russula flavida* Frost ex Peck			√		√	√	EM	Poisonous	Agarics	
*Russula foetens* Pers.	√		√	√	√	√	EM	Medicinal, Poisonous	Agarics	ON683488
*Russula fragilis* Fr.	√		√				EM	Medicinal, Poisonous	Agarics	ON683489
*Russula furcata* Pers.			√				EM	Edible	Agarics	
*Russula grata* Britzelm.			√		√	√	EM	Others	Agarics	
*Russula integra* (L.) Fr.	√		√				EM	Edible, Medicinal	Agarics	
*Russula lilacea* Quél.	√			√			EM	Edible, Medicinal	Agarics	
*Russula mariae* Peck						√	EM	Edible	Agarics	
*Russula mustelina* Fr.	√				√		EM	Edible	Agarics	
*Russula nauseosa* (Pers.) Fr.	√		√				EM	Edible	Agarics	
*Russula paludosa* Britzelm.			√	√			EM	Edible	Agarics	
*Russula pectinata* Fr.					√		EM	Poisonous	Agarics	
*Russula pseudodelica* J.E. Lange	√		√				EM	Edible, Medicinal	Agarics	
*Russula puellaris* Fr.	√		√				EM	Edible	Agarics	
*Russula pungens* Beardslee						√	EM	Others	Agarics	
*Russula risigallina* (Batsch) Sacc.	√			√			EM	Edible	Agarics	
*Russula rosea* Pers.	√						EM	Edible, Medicinal	Agarics	
*Russula rubra* (Lam.) Fr.	√		√				EM	Edible	Agarics	
*Russula sanguinaria* (Schumach.) Rauschert	√		√	√			EM	Edible	Agarics	
*Russula sanguinea* Fr.	√				√	√	EM	Others	Agarics	
*Russula sororia* (Fr.) Romell				√	√	√	EM	Medicinal	Agarics	ON683431
*Russula squalida* Peck				√			EM	Others	Agarics	
*Russula subdepallens* Peck	√		√				EM	Edible	Agarics	
*Russula vinosa* Lindblad				√			EM	Others	Agarics	ON683432
*Sarcodontia spumea* (Sowerby) Spirin	√	√	√	√	√		EM	Others	PHT fungi	
*Sarcomyxa edulis* (Y.C. Dai, Niemelä, and G.F. Qin) T. Saito, Tonouchi, and T. Harada	√	√			√		WS	Medicinal	Agarics	
*Sarcoscypha coccinea* (Gray) Boud.		√	√	√	√		WS	Poisonous	Larger Ascomycetes	
*Schizophyllum commune* Fr.	√	√	√	√	√	√	WS	Edible, Medicinal	Agarics	
*Scleroderma areolatum* Ehrenb.		√	√			√	SS	Edible, Medicinal, Poisonous	Gasteroid fungi	
*Scleroderma bovista* Fr.		√	√		√		SS	Edible, Medicinal	Gasteroid fungi	
*Scleroderma polyrhizum* (J.F. Gmel.) Pers.					√		SS	Edible, Medicinal	Gasteroid fungi	
*Scutellinia pseudovitreola* W.Y. Zhuang and Zhu L. Yang		√					WS	Others	Larger Ascomycetes	
*Scutellinia scutellata* (L.) Lambotte	√	√	√		√	√	WS	Others	Larger Ascomycetes	
*Sparassis latifolia* Y.C. Dai and Zheng Wang		√					WS	Edible, Medicinal	PHT fungi	ON683490
*Spathularia flavida* Pers.				√	√	√	SS	Others	Larger Ascomycetes	
*Sphaerobolus stellatus* Tode	√					√	WS	Others	Gasteroid fungi	
*Spongiporus zebra* (Y.L. Wei and W.M. Qin) B.K. Cui, L.L. Shen, and Y.C. Dai	√					√	WS	Others	PHT fungi	
*Steccherinum ochraceum* (Pers. ex J.F. Gmel.) Gray			√				WS	Others	PHT fungi	
*Steccherinum rawakense* (Pers.) Banker	√			√			WS	Others	PHT fungi	
*Stereum hirsutum* (Willd.) Pers.	√	√			√	√	WS	Medicinal	PHT fungi	
*Stereum rugosum* Pers.	√				√		WS	Others	PHT fungi	
*Stereum subtomentosum* Pouzar		√					WS	Others	PHT fungi	
*Stereum ostrea* (Blume and T. Nees) Fr.	√	√	√		√		WS	Others	PHT fungi	
*Strobilurus stephanocystis* (Kühner and Romagn. ex Hora) Singer						√	WS	Others	Agarics	
*Stropharia aeruginosa* (Curtis) Quél.		√	√		√		SS	Edible, Poisonous	Agarics	
*Stropharia rugosoannulata* Farl. ex Murrill	√	√			√		SS	Edible, Medicinal	Agarics	
*Suillellus luridus* (Schaeff.) Murrill					√		EM	Edible, Medicinal	Boletes	
*Suillus bovinus* (L.) Roussel					√	√	EM	Edible, Medicinal, Poisonous	Boletes	
*Suillus flavus* (Quél.) Singer	√				√		EM	Others	Boletes	
*Suillus granulatus* (L.) Roussel	√		√	√	√	√	EM	Edible, Medicinal, Poisonous	Boletes	ON683433
*Suillus grevillei* (Klotzsch) Singer	√			√	√	√	EM	Edible, Medicinal	Boletes	
*Suillus lactifluus* (With.) A.H. Sm. and Thiers						√	EM	Edible	Boletes	
*Suillus laricinus* (Berk.) Kuntze					√	√	EM	Others	Boletes	
*Suillus luteus* (L.) Roussel	√				√		EM	Edible, Medicinal, Poisonous	Boletes	
*Suillus spraguei* (Berk. and M.A. Curtis) Kuntze	√						EM	Others	Boletes	
*Suillus subaureus* (Peck) Snell			√				EM	Edible, Medicinal	Boletes	
*Suillus viscidus* (L.) Roussel	√				√		EM	Edible, Medicinal	Boletes	
*Tapinella atrotomentosa* (Batsch) Šutara			√		√	√	EM	Medicinal, Poisonous	Boletes	
*Tapinella panuoides* (Fr.) E.-J. Gilbert		√		√			EM	Poisonous	Boletes	
*Terana caerulea* (Lam.) Kuntze			√			√	WS	Others	PHT fungi	
*Tetrapyrgos nigripes* (Fr.) E. Horak	√	√				√	WS	Others	Agarics	
*Thelephora anthocephala* (Bull.) Fr.			√	√			SS	Others	PHT fungi	
*Thelephora palmata* (Scop.) Fr.					√	√	SS	Others	PHT fungi	
*Tolypocladium capitatum* (Holmsk.) C.A. Quandt, Kepler, and Spatafora						√	EI	Others	Larger Ascomycetes	
*Trametes coccinea* (Fr.) Hai J. Li and S.H. He		√	√				WS	Others	PHT fungi	
*Trametes conchifer* (Schwein.) Pilát		√		√			WS	Others	PHT fungi	
*Trametes gibbosa* (Pers.) Fr.	√				√		WS	Medicinal	PHT fungi	ON683491
*Trametes hirsuta* (Wulfen) Lloyd			√		√	√	WS	Medicinal	PHT fungi	
*Trametes membranacea* (Sw.) Kreisel						√	WS	Others	PHT fungi	
*Trametes pubescens* (Schumach.) Pilát	√			√			WS	Edible, Medicinal, Poisonous	PHT fungi	
*Trametes suaveolens* (L.) Fr.	√				√	√	WS	Medicinal	PHT fungi	
*Trametes trogi* Berk.					√		WS	Poisonous	PHT fungi	
*Trametes versicolor* (L.) Lloyd	√	√	√	√		√	WS	Medicinal	PHT fungi	ON683492
*Tremella aurantia* Schwein.					√		WS	Edible	Jelly fungi	
*Tremella foliacea* Pers.		√					WS	Others	Jelly fungi	ON683493
*Tremella fuciformis* Berk.		√	√				WS	Edible, Medicinal	Boletes	
*Tremella mesenterica* (Schaeff.) Pers.	√	√				√	WS	Edible, Medicinal	Jelly fungi	
*Trichaptum abietinum* (Pers. ex J.F. Gmel.) Ryvarden	√			√			WS	Medicinal	PHT fungi	
*Trichaptum biforme* (Fr.) Ryvarden			√				WS	Medicinal	PHT fungi	
*Trichaptum pargamenum* (Fr.) G. Cunn.		√				√	WS	Others	PHT fungi	
*Tricholoma acerbum* (Bull.) Quél.	√						EM	Edible, Medicinal, Poisonous	Agarics	
*Tricholoma album* (Schaeff.) P. Kumm.	√		√				EM	Edible, Medicinal, Poisonous	Agarics	
*Tricholoma aurantium* (Schaeff.) Ricken	√				√		EM	Others	Agarics	
*Tricholoma equestre* (L.) P. Kumm.				√			EM	Edible, Poisonous	Agarics	
*Tricholoma matsutake* (S. Ito and S. Imai) Singer	√	√					EM	Edible, Medicinal	Agarics	
*Tricholoma scalpturatum* (Fr.) Quél.				√			EM	Edible, Poisonous	Agarics	
*Tricholoma terreum* (Schaeff.) P. Kumm.			√		√	√	EM	Others	Agarics	
*Tricholoma tigrinum* (Schaeff.) Gillet	√			√			EM	Poisonous	Agarics	
*Tricholoma vaccinum* (Schaeff.) P. Kumm.	√			√			EM	Edible, Medicinal	Agarics	
*Tricholomopsis decora* (Fr.) Singer	√						WS	Edible	Agarics	
*Tricholomopsis rutilans* (Schaeff.) Singer	√	√	√	√	√	√	WS	Poisonous	Agarics	
*Tulostoma bonianum* Pat.			√		√	√	SS	Others	Gasteroid fungi	
*Turbinellus floccosus* (Schwein.) Earle ex Giachini and Castellano			√	√			EM	Poisonous	Cantharelloid fungi	
*Verpa bohemica* (Krombh.) J. Schröt.						√	SS	Edible, Medicinal, Poisonous	Larger Ascomycetes	
*Verpa digitaliformis* Pers.						√	SS	Edible	Larger Ascomycetes	
*Vitreoporus dichrous* (Fr.) Zmitr.					√		WS	Others	PHT fungi	
*Volvariella bombycina* (Schaeff.) Singer			√		√		WS	Edible, Medicinal	Agarics	
*Volvariella pusilla* (Pers.) Singer						√	SS	Edible, Medicinal	Agarics	
*Volvopluteus gloiocephalus* (DC.) Vizzini, Contu and Justo			√		√		SS	Poisonous	Agarics	
*Xanthochrous gilvicolor* (Lloyd) Teng	√					√	WS	Others	PHT fungi	
*Xerocomellus chrysenteron* (Bull.) Šutara					√		EM	Others	Boletes	
*Xerocomus chrysenteron* (Bull.) Quél.	√				√	√	EM	Others	Boletes	
*Xeromphalina campanella* (Batsch) Kühner and Maire	√	√	√	√	√		WS	Medicinal	Agarics	
*Xerula pudens* (Pers.) Singer	√						WS	Others	Agarics	
*Xylaria carpophila* (Pers.) Fr.			√			√	WS	Medicinal	Larger Ascomycetes	
*Xylaria hypoxylon* (L.) Grev.	√	√	√		√	√	WS	Others	Larger Ascomycetes	
*Xylaria polymorpha* (Pers.) Grev.		√	√	√			WS	Others	Larger Ascomycetes	

Note: EM = ectomycorrhizal; SS = soil saprotroph; WS = wood saprotroph; LS = litter saprotroph; DS = dung saprotroph; EI = endophyte insect pathogen.

**Table A2:** Species scientific names and their corresponding abbreviations.

Abbreviation	Genus	Abbreviation	Genus	Abbreviation	Genus	Abbreviation	Genus
Abo	*Abortiporus*	Dac	*Dacrymyces*	Lec	*Leccinum*	Pip	*Piptoporus*
Aga	*Agaricus*	Dacr	*Dacryopinax*	Len	*Lentinellus*	Ple	*Pleurotus*
Agr	*Agrocybe*	Dae	*Daedalea*	Lent	*Lentinus*	Plu	*Pluteus*
Ale	*Aleuria*	Daed	*Daedaleopsis*	Lenz	*Lenzites*	Pol	*Polyporus*
Ama	*Amanita*	Dal	*Daldinia*	Leo	*Leotia*	Poly	*Polystictus*
Amp	*Ampulloclitocybe*	Dec	*Deconica*	Lep	*Lepiota*	Pos	*Postia*
Api	*Apioperdon*	Des	*Descolea*	Lepi	*Lepista*	Psa	*Psathyrella*
Arm	*Armillaria*	Dum	*Dumontinia*	Leu	*Leucoagaricus*	Pse	*Pseudoclitocybe*
Art	*Artomyces*	Ent	*Entoloma*	Leuc	*Leucocybe*	Pseu	*Pseudosperma*
Asc	*Ascocoryne*	Exi	*Exidia*	Lyc	*Lycoperdon*	Pte	*Pterula*
Aur	*Auricularia*	Flam	*Flammulaster*	Lyo	*Lyophyllum*	Pyc	*Pycnoporus*
Auri	*Auriscalpium*	Flamm	*Flammulina*	Lys	*Lysurus*	Rad	*Radulodon*
Bis	*Bisporella*	Fom	*Fomes*	Mac	*Macrocystidia*	Ram	*Ramaria*
Bje	*Bjerkandera*	Fomi	*Fomitopsis*	Macr	*Macrolepiota*	Res	*Resupinatus*
Bol1	*Boletinellus*	Gal	*Galerina*	Mar	*Marasmiellus*	Rho	*Rhodocollybia*
Bol	*Boletus*	Gan	*Ganoderma*	Mara	*Marasmius*	Ric	*Rickenella*
Cal1	*Calocera*	Gea	*Geastrum*	Mel	*Melanoleuca*	Rus	*Russula*
Calo	*Calocybe*	Ger	*Gerronema*	Mor	*Morchella*	Sar	*Sarcodontia*
Cal	*Calvatia*	Glo	*Gloeophyllum*	Muc	*Mucidula*	Sar	*Sarcomyxa*
Can	*Cantharellus*	Gloe	*Gloeostereum*	Mut	*Mutinus*	Sarc	*Sarcoscypha*
Cer	*Cerioporus*	Gue	*Guepinia*	Myc	*Mycena*	Sch	*Schizophyllum*
Che	*Cheilymenia*	Gym	*Gymnopilus*	Neo	*Neofavolus*	Scl	*Scleroderma*
Chl	*Chlorociboria*	Gymn	*Gymnopus*	Neol	*Neolentinus*	Scu	*Scutellinia*
Chr	*Chroogomphus*	Har	*Harrya*	Omp	*Omphalotus*	Spa	*Spathularia*
Cla	*Clavaria*	Hel	*Helvella*	Oph	*Ophiocordyceps*	Ste	*Steccherinum*
Clav	*Clavariadelphus*	Hem	*Hemistropharia*	Oss	*Ossicaulis*	Ste	*Stereum*
Clavu	*Clavulina*	Her	*Hericium*	Oti	*Otidea*	Str	*Stropharia*
Clavul	*Clavulinopsis*	Het	*Heterobasidion*	Pan	*Panaeolus*	Sui	*Suillus*
Cli	*Clitocybe*	Hoh	*Hohenbuehelia*	Pane	*Panellus*	Tap	*Tapinella*
Col	*Coltricia*	Hum	*Humaria*	Panu	*Panus*	Ter	*Terana*
Con	*Connopus*	Hyd	*Hydnum*	Par	*Paralepista*	The	*Thelephora*
Cono	*Conocybe*	Hyg	*Hygrocybe*	Para	*Parasola*	Tra	*Trametes*
Cop	*Coprinellus*	Hygr	*Hygrophorus*	Pax	*Paxillus*	Tre	*Tremella*
Copr	*Coprinopsis*	Hym	*Hymenopellis*	Per	*Perenniporia*	Tri	*Trichaptum*
Copri	*Coprinus*	Hyp	*Hypholoma*	Pez	*Peziza*	Tric	*Tricholoma*
Cor	*Cordyceps*	Hyp	*Hypsizygus*	Pha	*Phaeolepiota*	Trich	*Tricholomopsis*
Cori	*Coriolopsis*	Inf	*Infundibulicybe*	Phae	*Phaeotremella*	Tul	*Tulostoma*
Cort	*Cortinarius*	Ino	*Inocybe*	Pha	*Phallus*	Tur	*Turbinellus*
Cot	*Cotylidia*	Ino	*Inonotus*	Phe	*Phellinus*	Vol	*Volvariella*
Cre	*Crepidotus*	Irp	*Irpex*	Phel	*Phellodon*	Volv	*Volvopluteus*
Cup	*Cuphophyllus*	Kue	*Kuehneromyces*	Phl	*Phloeomana*	Xer1	*Xerocomus*
Cya	*Cyathus*	Lac	*Laccaria*	Pho	*Pholiota*	Xer	*Xeromphalina*
Cyc	*Cyclocybe*	Lact	*Lactarius*	Phy	*Phyllotopsis*	Xyl	*Xylaria*
Cys	*Cystoderma*	Lae	*Laetiporus*	Pic	*Picipes*		
